# An automated method for the assessment of the rice grain germination rate

**DOI:** 10.1371/journal.pone.0279934

**Published:** 2023-01-03

**Authors:** Yongzhong Zhang, Hexiao Huang, Binbin Xiong, Yan Ma

**Affiliations:** 1 School of Science and Technology, Shanghai Open University, Shanghai, China; 2 College of Information, Mechanical and Electrical Engineering, Shanghai Normal University, Shanghai, China; China National Rice Research Institute, CHINA

## Abstract

The germination rate of rice grain is recognized as one of the most significant indicators of seed quality assessment. Currently, grain germination rate is generally determined manually by experienced researchers, which is time-consuming and labor-intensive. In this paper, a new method is proposed for counting the number of grains and germinated grains. In the coarse segmentation process, the k-means clustering algorithm is applied to obtain rough grain-connected regions. We further refine the segmentation results obtained by the k-means algorithm using a one-dimensional Gaussian filter and a fifth-degree polynomial. Next, the optimal single grain area is determined based on the area distribution curve. Accordingly, the number of grains contained in the connected region is equal to the area of the connected region divided by the optimal single grain area. Finally, a novel algorithm is proposed for counting germinated grains. This algorithm is based on the idea that the length of the intersection between the germ and the grain is less than the circumference of the germ. The experimental results show that the mean absolute error of the proposed method for germination rate is 2.7%. And the performance of the proposed method is robust to changes in grain number, grain varieties, scale, illumination, and rotation.

## Introduction

Rice is one of the three most important food crops, and its quality mainly depends on the quality of the seeds. In agricultural production, the germination rate of grains is often used as one of the important indicators for judging seed quality. Therefore, an accurate assessment of grain germination rate will help to accurately assess seed quality.

In the process of multi-batch measurement, the seed germination rate is generally determined manually by experienced researchers, in which the number of grains and the germinated grains are counted separately. This work is time-consuming and laborious, and the standard for determining the germination of grains is susceptible to human factors, which makes it difficult for different personnel to achieve consistent results and the experiment unrepeatable.

In recent years, the development of image processing and pattern recognition technology has provided conditions for the automatic quantitative assessment of seed germination rate. The grain germination rate assessment based on image processing can be divided into the following three steps: (1) grain segmentation; (2) grain counting; (3) germinated grain counting.

The segmentation of grains is to regard grains as the foreground and separate them from the background. In this way, a binary image can be used to represent the grain image, which provides convenience for subsequent grain counting. One of the difficulties of grain segmentation is that the color of the grain is easily affected by illumination changes. When the illumination is strong, the color of the grain tends to be white. On the contrary, when the illumination is dark, the color of the grain tends to be gray. To reduce the illumination influence, Wu et al. [1] transformed the image from RGB color space to Lab color space, used two color difference components *a* and *b* to represent pixels, and then exploited k-means clustering algorithm for grain segmentation. The segmentation effect of this method is affected by the initial clustering center, and the randomly selected initial clustering center may make the final segmentation result wrong. In addition, active contour model is often used for grain segmentation [2].

Most grains appear as a cluster of multiple touching grains in the grain image. If we can remove the adhesions between the grains while separating the grains from the image, the number of grains can be expressed in terms of the number of connected regions in the binary image. The watershed-based segmentation method [3] can perform grain segmentation, but it is prone to over-segmentation when there is noise and rough edges in the image. In contrast, serious adhesions between grains can cause the under-segmentation problem. Some researchers propose to combine the watershed algorithm with other methods to solve the above problems. Duarte et al. [4] used an undirected graph to represent the segmentation results after watershed transformation and used a hierarchical social metaheuristic to improve image segmentation quality. Wang et al. [5] constructed the internal markers through a series of morphological operations to solve the over-segmentation problem in watershed transformation. Yang et al. [6] incorporated gray-scale gradient information into the watershed transformation to segment the overlapping objects.

The touching grains generally form concave points at the adhesive part between the grains, and the segmentation line formed by the concave point pair can separate the grains [7]. Generally, the concave point can be detected based on the curvature of the grain contour and the corner points. However, rough edges will seriously affect the curvature of the contour, which will cause errors in concave point detection. To accurately detect the concave points, Mebatsion et al. [8] used an elliptic Fourier series approximation to smooth the boundary contours of the image. In addition, Lin et al. [9] smoothed the contour curve by convolving the curve with a Gaussian kernel function. Liu et al. [10] used a circular template with a fixed radius to move along the contour and detect the concave points based on the response value. However, a circular template with a fixed radius is likely to result in a missed or wrong detection. In addition, another difficulty in the separation process is concave point matching. The currently proposed matching rules for concave point pairs include the nearest-neighbor criterion [11] and the radian critical distance criterion [12].

The initial method used for seed germination detection is to take the original grain image as a template and compare the germinated grain with the ungerminated grain [13]. In this method, the camera position is required to be fixed and the seeds need to be neatly arranged in equal intervals with adhesions. And the image acquisition system is not conducive to popularization due to many restrictions. Mladenov et al. [14] proposed to assess the seed germination with neural network. And the seeds are also not allowed to touch each other. The seeds with roots are regarded as germinated seeds in the method proposed by Khoenkaw [15]. In this method, the distance between the camera and the seed needs to be fixed and whether there is a germ is determined by a preset threshold, which also brings inconvenience to practical application. Recently, convolutional neural networks have been proposed and applied to classify objects, such as SSD [16], YOLO [17], R-CNN [18], and Fast R-CNN [18]. Genze et al. [19] used Fast R-CNN to distinguish whether the grains have germinated. To facilitate labeling, these methods require that the grains in the training images or test images are independent of each other, which also brings inconvenience to actual use.

The most commonly used methods including watershed algorithm, concave point detection, and active contour model, try to separate the adhered grains to obtain the accurate grain number. However, grains touch each other in a complex pattern and the intersection between the adhesion grains forms different strong or weak edges. And these methods are not applicable in all complex adhesion situations. To solve this problem, the basic idea of the proposed algorithm is as follows:

Assuming that we separate all the grains from the image and use a binary image to represent the grains, in which either isolated grains or adhesion grains can be regarded as the connected regions with different areas. If the area of a single grain is denoted as *s*_0_, the number of grains contained in the connected region is equal to the area of the connected region divided by *s*_0_. Then we can obtain the total number of grains by adding the grain number contained in all connected regions. The advantage of this is that the operation of removing the adhesion between the grains can be avoided.

In addition, most of the aforementioned methods of grain germination detection require a fixed camera position and the grains keep a certain distance from each other without any adhesion. But it is difficult to meet such requirements in actual use. The basic idea of grain germination detection in this paper is as follows:

We can further separate the grain germ (or radicle) part according to the color difference between the yellow grain and the white germ (or radicle). Then, the germs (or radicles) are further selected according to the following characteristics: (1) There is a certain proportional relationship between the area of the germ (or radicle) and the area of a single grain *s*_0_; (2) The germ (or radicle) must be attached to the grain; (3) The length of the intersection between the germ (or radicle) and the grain is less than the circumference of the germ (or radicle).

The rice grain germination rate assessment system proposed in this paper has low requirements in the experimental environment. It can be used for shooting at any time with a mobile phone. The server completes the assessment of the germination rate and returns the results to the mobile terminal. The whole process takes about 1 to 2 seconds.

## Materials and methods

### Test materials

First, prepare a piece of less reflective black paper as the background. The black background has a large difference in color in comparison to the yellow grains and white germs (or radicles), which is convenient for subsequent recognition. Next, prepare a less reflective black container with some water and grains. If the container is highly reflective, it will interfere with subsequent grain recognition. Lastly, the containers are placed in a thermostat for dark cultivation at 25°C and photographed after 24 to 48 hours.

### Software development tools

**Fig 1:**
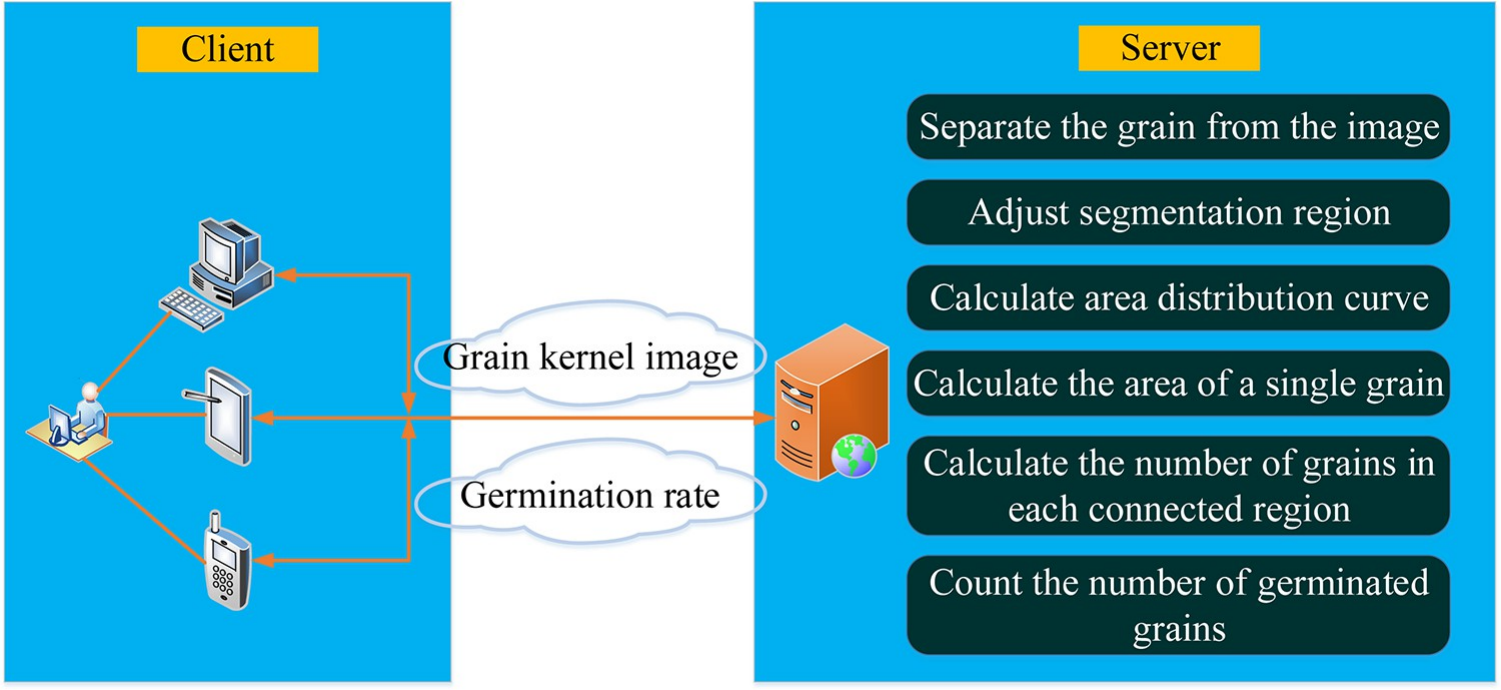
The system diagram.

We develop web applications in Python. Users can access the URL (http://117.68.114.143:82/) with a browser through the computer, laptop computer, or mobile phone, select the image to be recognized and upload it to the server. And the server runs the recognition algorithm, and finally, the data such as germination rate is sent back to the client. The results of numerous experiments demonstrate that the proposed algorithm in this paper is robust to variation in scale, rotation, and illumination. The system diagram is shown in Fig 1.

### Image capture

When capturing an image, the user can place the phone on top of the container and keep the phone as horizontal as possible. Only the black background and the container are included in the captured image. The distance between the phone and the container can be adjusted up and down as needed.

### Coarse segmentation

**Fig 2:**
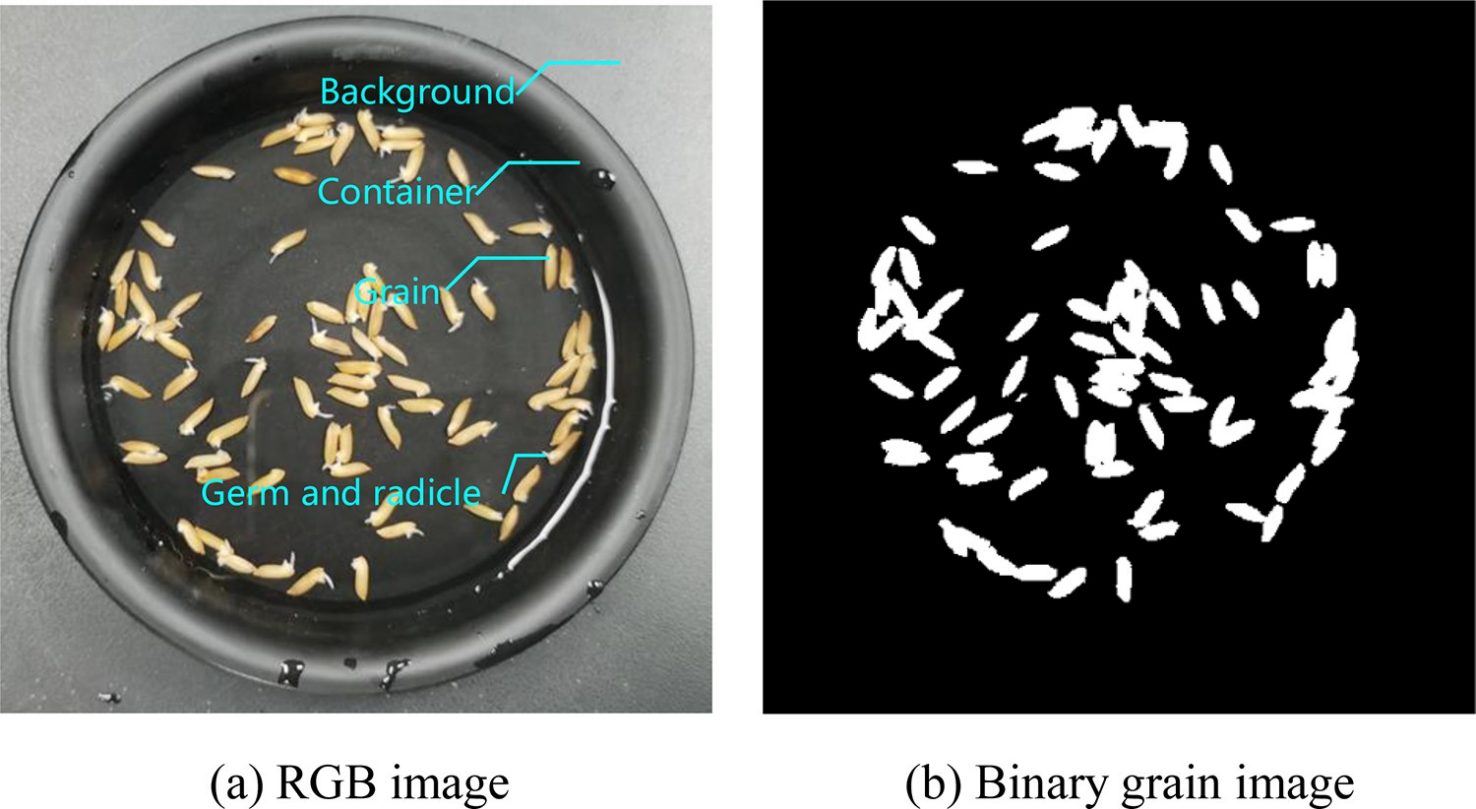
Coarse segmentation.

In coarse segmentation, we need to segment the grains from the image and convert the RGB color image into a binary grain image. As shown in Fig 2A, the RGB image includes black background and the container. The germ (or radicle) is white and the grains are yellow in the container. Because there is a certain amount of water in the container, the water appears white under the light. According to the additive color mixing theory of light, yellow is mixed with red and green. We denote the *R*, *G*, and *B* values of the pixel at position (*x*,*y*) in the image by *R*(*x*,*y*), *G*(*x*,*y*), and *B*(*x*,*y*), respectively. When both the values of *R*(*x*,*y*)−*B*(*x*,*y*) and *G*(*x*,*y*)−*B*(*x*,*y*) are larger, the corresponding pixel color is yellow, otherwise, it is black or white. The distribution of *R*(*x*,*y*)−*B*(*x*,*y*) and *G*(*x*,*y*)−*B*(*x*,*y*) of the grains is quite different from each other due to different illumination and grain soaking time. Therefore, the grains cannot be correctly segmented by threshold. In this paper, k-means clustering algorithm [20] is used for grain coarse segmentation. First, represent the attributes of pixel by *r*_*b*_ and *g*_*b*_.


$$r_{b}=\{\begin{array}{l} \;30\;R-B\geq 60\\ R-B\;\mathrm{otherwise} \end{array}$$ (1)

$$g_{b}=\{\begin{array}{c} \;30\;G-B\geq 60\\ G-B\;\mathrm{otherwise} \end{array}$$ (2)

K-means algorithm can classify the pixels according to the distance between each pixel and the cluster center. Since the *R*−*B* span is large and the color of the pixels on the border of the grain is close to white or black, when *K* = 2, the border pixels of the grain may be wrongly classified as background. To avoid this error, we reduce the *R*−*B* span by Eq (1). The *r*_*b*_ value is uniformly set to 30 when the *R*−*B* value is greater than or equal to 60, otherwise, it remains unchanged. The same is true for the *g*_*b*_ value. Algorithm 1 lists the clustering process of the k-means algorithm. Two initial cluster centers are fixed at (0,0) and (40,40) respectively to avoid the effect of a random selection of the initial cluster centers on the clustering effect.

    Algorithm 1 K-means algorithm

Input: *K* = 2(number of clusters), Dataset *P* = {*p*_1_ ($r_{b}^{1}$, $g_{b}^{1}$), *p*_2_ ($r_{b}^{2}$, $g_{b}^{2}$), …,*p*_n_ ($r_{b}^{n}$, $g_{b}^{n}$)}

    Two initial cluster centers *C* = {*c*_1_(0,0), *c*_2_(40,40)}

Output: A set of *K* clusters.

1 repeat

2    for *i* = 1:*n*

3        find the closest center *c*_*k*_∈*C* to pixel *p*_*i*_ ($r_{b}^{i}$, $g_{b}^{i}$)

4            assign pixel *p*_*i*_ to cluster *C*_*k*_

5    endfor

6    Compute the new centroid (mean) of each cluster

7 until the centroid positions do not change

Fig 2B shows the binary grain image after applying k-means algorithm on the grain image as shown in Fig 2A. The connected regions which are constituted by the grains are called grain connected regions. It can be seen from Fig 2B that the background, container, the white reflection part on the container, and the germ (or radicle) are all classified into the same category.

### Refinement segmentation

**Fig 3:**
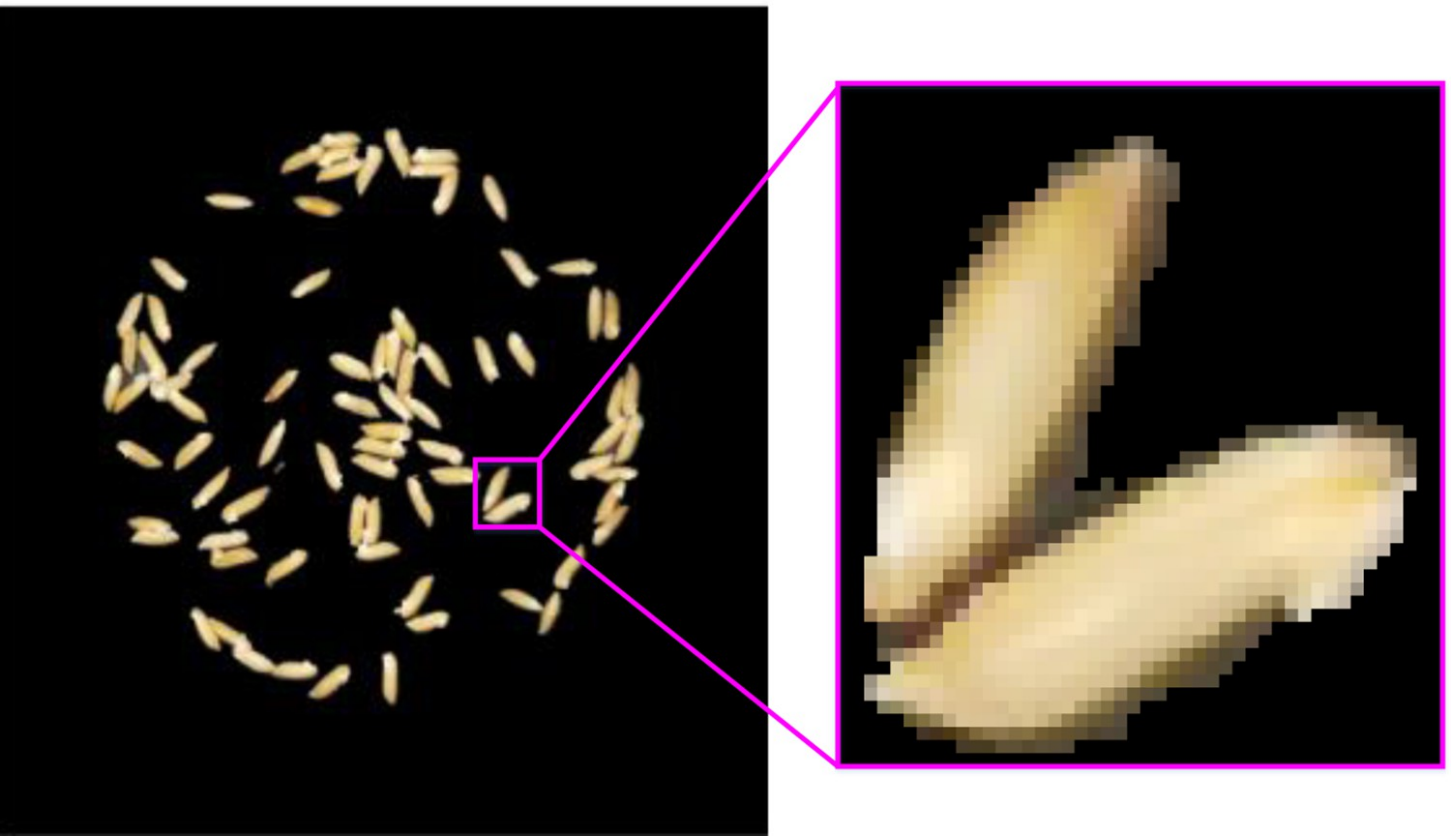
Magnified image.

Fig 3 shows the image by combining Fig 2A and 2B, that is, the white part (grain connected regions) in the binary image is represented by the corresponding pixel color in the RGB image. To further observe the coarse segmentation results, we magnify the grain in the purple rectangle, as shown in Fig 3. We can see from Fig 3 that the grain boundaries are relatively rough after coarse segmentation, that is, the separated foreground grains include the core part of the grain (bright area) and the border part of the grain (dark domain). This is mainly due to the fact that there exist shadows around the grain boundaries and the color of the shadow is closer to that of the yellow grain. Hence the shadows are classified as part of the grains in k-means clustering. Therefore, the clustering results of k-means need to be refined.

**Fig 4:**
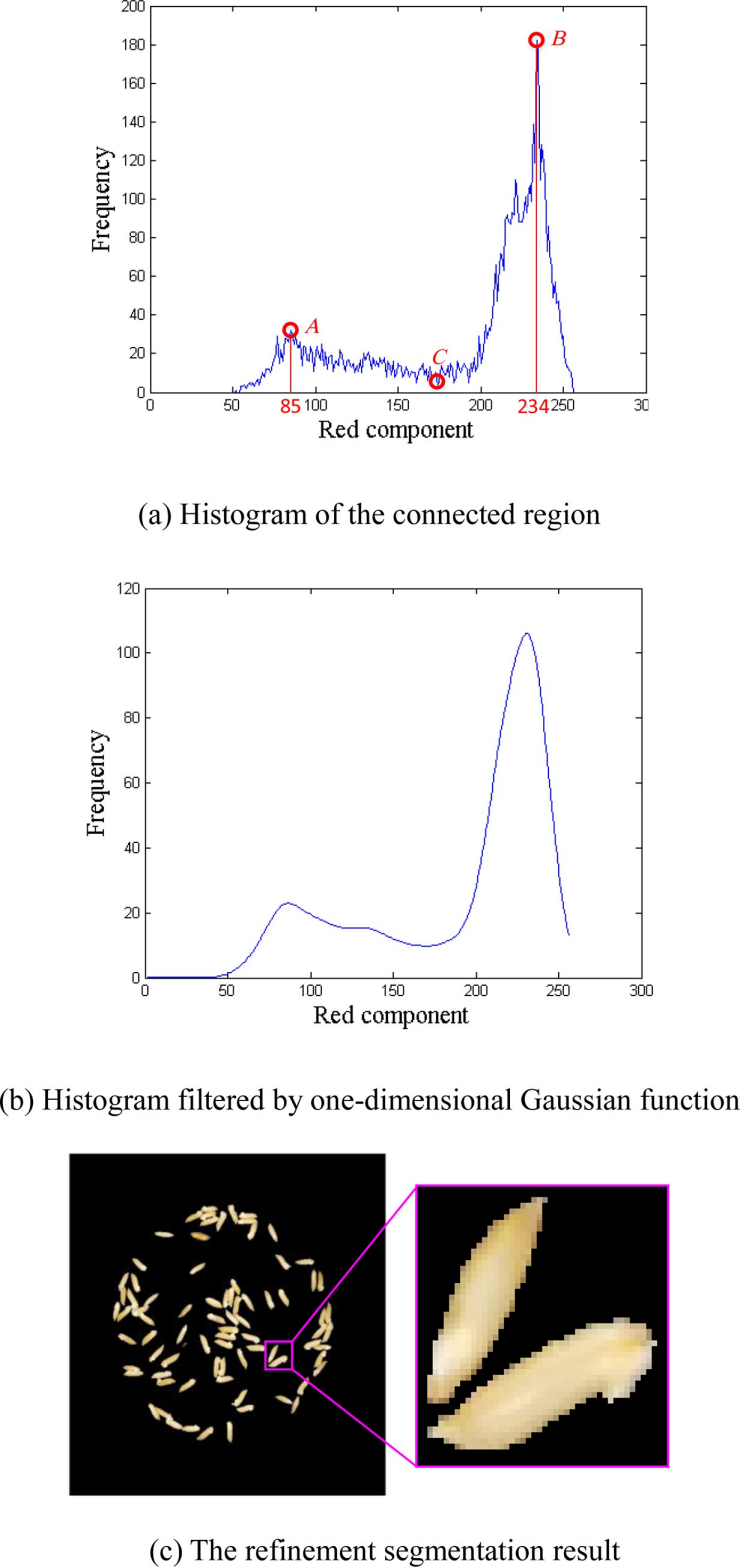
Refinement segmentation.

Fig 4A shows the histogram of the red component of the grain part in the magnified image of Fig 3. It can be seen from Fig 4A that there are one valley and two peaks in the histogram. The border part of the grain corresponds to peak point *A* whose red component value is 85. And the core part of the grain corresponds to peak point *B* whose red component value is 234. There is a valley point *C* between the two peak points *A* and *B*. The red component value of point *C* can be regarded as the threshold *d* separating the core part and border part of the grain. In the refinement segmentation stage, the pixels in the grain connected region whose red component value is less than *d* are further classified into the background.

Because the histogram shown in Fig 4A is not smooth, it is difficult to locate the valley point directly from the original histogram. We use a one-dimensional Gaussian filter [21] to filter the histogram. Fig 4B shows the histogram filtered by a Gaussian filter. The Gaussian filter is defined as:

$$G(x)=\exp(-x^{2}/2\sigma^{2})$$ (3)

where *σ* is the standard deviation.

The fluctuation of the histogram curve cannot be reduced in most cases if we use a smaller filter size. As a consequence, too many valley points will be detected, which will lead to wrong segmentation. Hence, we use a Gaussian filter of size 100 with a standard deviation of 7.5 in this paper.

**Fig 5:**
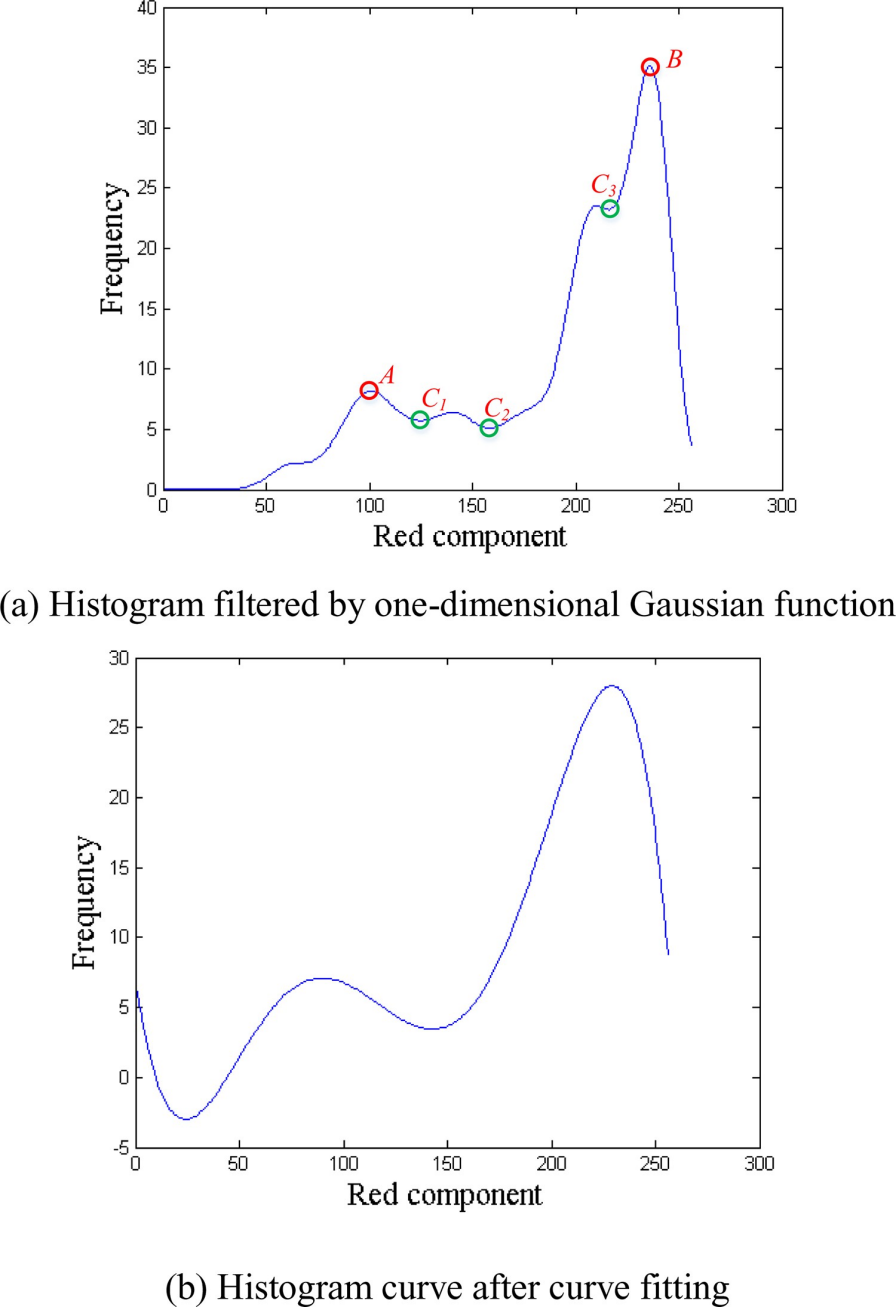
An example of a histogram.

After applying a Gaussian filter on a histogram, we found from experiments that there are multiple valley points in some histograms. As shown in Fig 5A, there are three valley points *C*_1_, *C*_2_, and *C*_3_ between the peak points *A* and *B*, which brings difficulties to the determination of the threshold. To further reduce the number of peak points and valley points, we perform curve fitting [22] on the smoothed histogram. Since we need to keep the true peak points and valley points, we select a 5th-degree polynomial to fit the histogram:

$$f(x)=ax^{5}+bx^{4}+cx^{3}+dx^{2}+ex+f$$ (4)

where *a*, *b*, *c*, *d*, *e*, and *f* are parameters whose values can be obtained according to the least-squares method.

Fig 5B shows the fitting result of the histogram curve shown in Fig 5A. It can be seen from Fig 5B that we can generate the trend line with 5th-degree polynomial curve fitting, in which some small fluctuations in the histogram curve can be neglected. Hence the position of the valley point between the two peak points can be easily determined according to the trend line.

### Area of single grain determination

After the refined segmentation, we obtain the grain connected regions of different sizes. The number of pixels contained in the connected region can be regarded as the area of the region, denoted as *s*. In addition, the area of a single grain is denoted as *s*_0_. When the grains have uniform grain size distribution and there is no occlusion between the grains, the number of grains in the connected region is equal to *s*/*s*_0_. Next, we need to determine *s*_0_.

**Fig 6:**
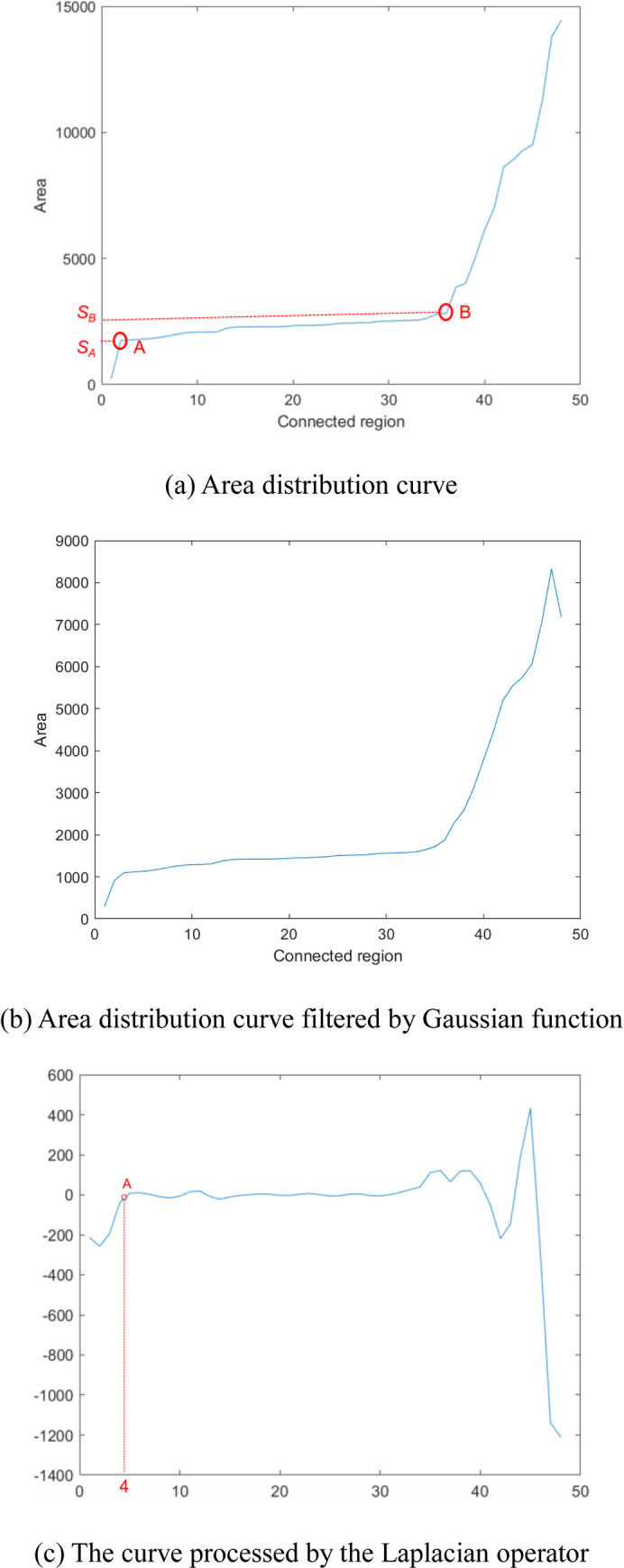
Determine the area of a single grain.

Assuming that a grain binary image *I*_*B*_ contains *m* grain connected regions. We sort the areas of *m* connected regions in ascending order. Fig 6A shows the area distribution curve after sorting. In Fig 6A, the area of the connected region located to the left of point *A* tends to be 0. These regions contain the noise in the image, which can be ignored when counting the number of grains. Points *A* and *B* in Fig 6A can be regarded as the turning points of the area distribution curve. It can be found that the curve between points *A* and *B* remains stable. Because there must be a part of connected regions composed of a single grain in the binary grain image, each connected region between *A* and *B* corresponds to the area of a single grain. The areas *s*_*A*_ and *s*_*B*_ for points *A* and *B* represent the minimum area and maximum area of all individual grains, respectively.

To determine the position of point *A*, we first use a one-dimensional Gaussian filter [23] with a length of 3 and σ of 0.65 to smooth the area distribution curve shown in Fig 6A. The result is shown in Fig 6B. Then one-dimensional Laplacian operator is applied on the smoothed curve, where the one-dimensional Laplacian operator can be expressed as:

$$\frac{\partial^{2}f}{\partial y^{2}}=f(x+1)+f(x-1)-2f(x)$$ (5)

where *f*() denotes the smoothed area distribution curve.

After the Laplacian operator processing, we obtain the new curve, denoted by *g*(*x*), as shown in Fig 6C. We start from the curve, the first point *g*(*i*) that satisfies the following condition can be considered as point *A*:

g(i−1)<0andg(i−1)<g(i)andg(i+1)>0andg(i+1)>g(i) (6)

Suppose that the area of the *i*th point on the area distribution curve is *s*_*i*_, correspondingly, the area of the (*i*+3)th point is s_*i*+3_. We start from point *A*, the point on the area distribution curve that satisfies the following condition can be regarded as the point *B*:

$$\frac{s_{i+3}}{s_{i}}>1.2$$ (7)

If point *B* that meets condition (7) cannot be found, it is considered that each grain connected region only contains a single grain, so the last point on the area distribution curve is taken as point *B*.

If *s*_*A*_ is taken as the area of a single grain, the obtained grain number will be larger than the true value since most grain area is larger than *s*_*A*_. In contrast, if *s*_*B*_ is taken as the area of a single grain, the obtained grain number will be smaller than the true value. And if we take the average value (*s*_*A*_+*s*_*B*_)/2 as the area of a single grain, it also cannot guarantee that the grain number is closest to the true value. To make the grain number closest to the true value, we propose Algorithm 2 to determine the optimal single grain area *s*_*opt*_.

    Algorithm 2 Determine the optimal single grain area

Input: A total of *m* candidate single grain areas between point *A* and point *B* {*s*_1_,*s*_2_,⋯,*s*_*m*_}

Output: The optimal single grain area *s*_*opt*_

1 *min_error* = 100000

2    for *i* = 1:*m*

3        *total_error* = 0 //Store the error caused by the *i*th single grain area

4        for *j* = 1:*m*

5            $total\_error=total\_error+abs(1‐\frac{s_{j}}{s_{i}})$

6        endfor

7        If *total_error*<*min_error*

8            *min_error* = *total_error*

9            *s*_*opt*_ = *s*_*i*_

10        endif

11 endfor

Assuming that the area of the *i*th connected region is *s*_*i*_ and the number of grains in this connected region is equal to $\left\lfloor \frac{s_{i}}{s_{opt}}\right\rfloor$. The result of $\frac{s_{i}}{s_{opt}}$ contains an integer part and a decimal part. If only the integer part is regarded as the number of grains in the connected region, the cumulative error will be accumulated when the number of grains contained in the connected region is large. To reduce the error, the number of grains in the connected region is equal to $\lfloor \frac{s_{i}}{s_{opt}}\rfloor +1$ when the decimal part is greater than the threshold 0.4, otherwise, the number of grains is equal to $\left\lfloor \frac{s_{i}}{s_{opt}}\right\rfloor$. In this way, the total number of grains is equal to the sum of the number of grains contained in each connected region.

### Germination detection

**Fig 7:**
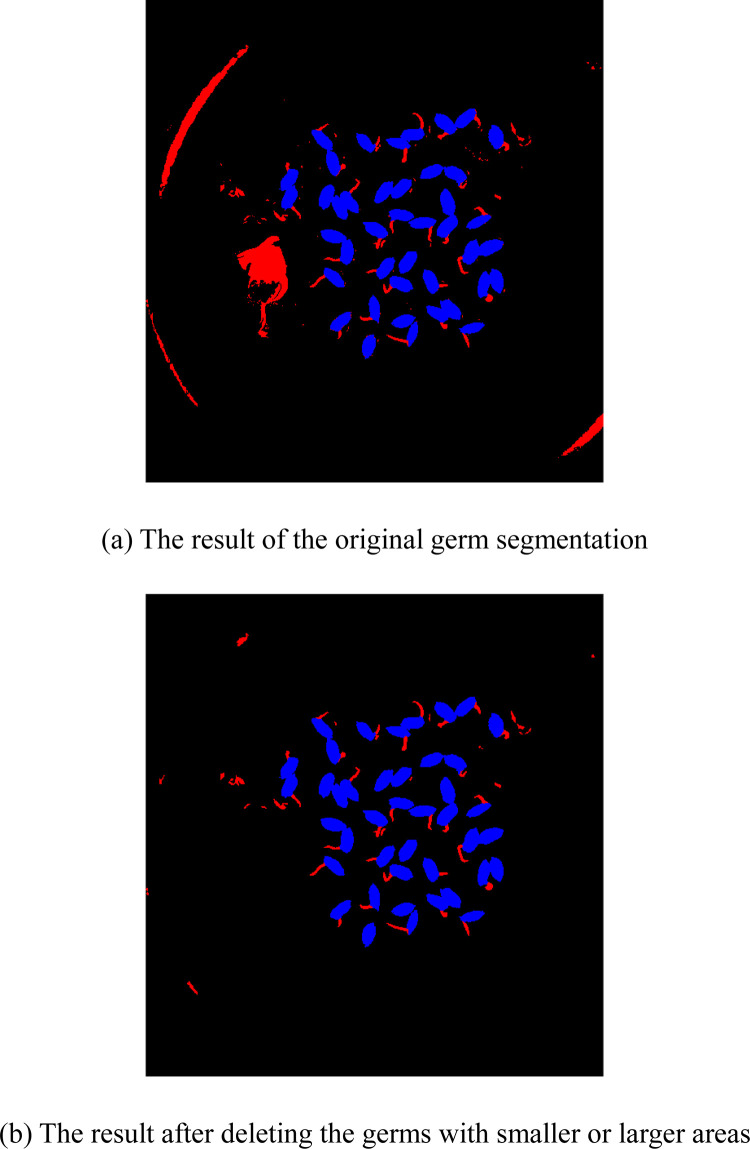
The result of germ segmentation.

We need to separate the germs from the grain image to further count the number of germinated grains. Since the germ is white, the pixel whose gray value is greater than 160 in the non-grain image can be considered as the germ, where the non-grain image represents the image after removing the grains. The red part in Fig 7A represents the germ connected region. It can be seen from Fig 7A that, in addition to the normal germ part, the reflective part in the image is considered as the germ. The area of germ connected region *s*_*bud*_ has a certain relationship with the single grain area *s*_*opt*_. When *s*_*bud*_ is much smaller than *s*_*opt*_, the germ is too small to see by the naked eye and it is not detected as a germ. In contrast, the germ is not counted if *s*_*bud*_ is much larger than *s*_*opt*_. Hence, we judge the germ according to the following condition after numerous experiments:

$$s_{bud}>\frac{s_{opt}}{50}\;\mathrm{and}\;s_{bud}<\frac{s_{opt}}{3}$$ (8)

**Fig 8:**
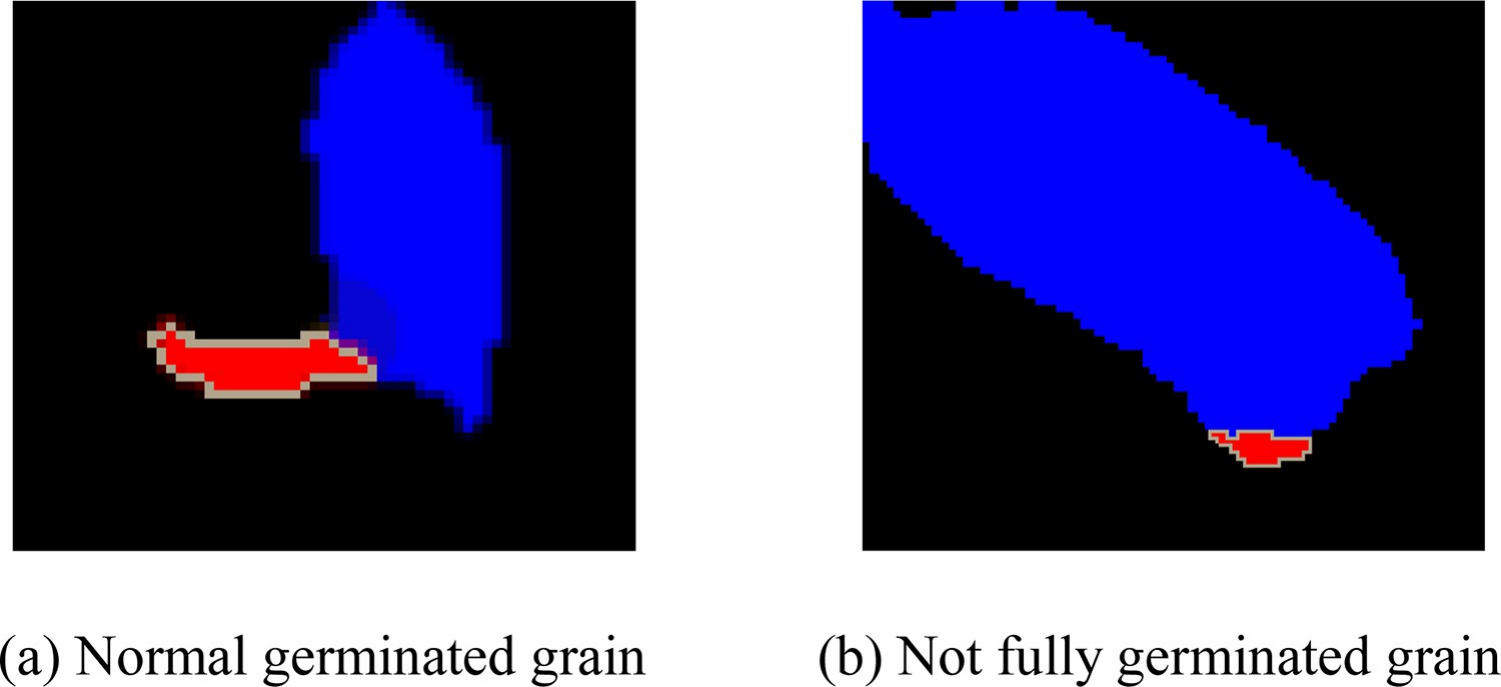
Example of germinated grain.

The germ connected region satisfying Eq (8) is regarded as a germ and the results are shown in Fig 7B. It can be seen from Fig 7B that most germs are extracted correctly, but there is still some noise not belonging to the germ. And some grains that have not fully germinated are predicted as germinated. Therefore, the extracted germs need to be further selected. Suppose that the length of the intersection part between the germ and grain is denoted by *l* and the circumference of the germ is denoted by *p*. There is a certain proportional relationship between *l* and *p*. As shown in Fig 8A, the normally germinated grain has a low value of *l*/*p*. As shown in Fig 8B, the grain that has not fully germinated has a larger value of *l*/*p*. Algorithm 3 represents the pseudo-code of selecting and counting the germs.

    Algorithm 3 Counting the number of germinated grains

Input: *m* grain connected regions $C_{g}=\{c_{g}^{1},c_{g}^{2},\cdots ,c_{g}^{m}\}$, *n* germ connected regions $\left\{c_{b}^{1},c_{b}^{2},\cdots ,c_{b}^{n}\right\}$, the circumferences of the germ connected regions $\left\{p_{b}^{1},p_{b}^{2},\cdots ,p_{b}^{n}\right\}$, the length of the intersection part between the germ and grain $\left\{l_{b}^{1},l_{b}^{2},\cdots ,l_{b}^{n}\right\}$.

Output: The number of germinated grains *n*_*bud*_

1    *n*_*bud*_ = 0

2    for *i* = 1:*n*

3        for *j* = 1:*m*

5            if $c_{b}^{i}$ and $c_{g}^{j}$ are adjacent and $l_{b}^{i}/p_{b}^{i}<0.4$

6                *n*_*bud*_ = *n*_*bud*_+1

7                break

8            endif

9        endfor

10    endfor

## Experiments and results

### Performance of grain counting method

**Fig 9:**
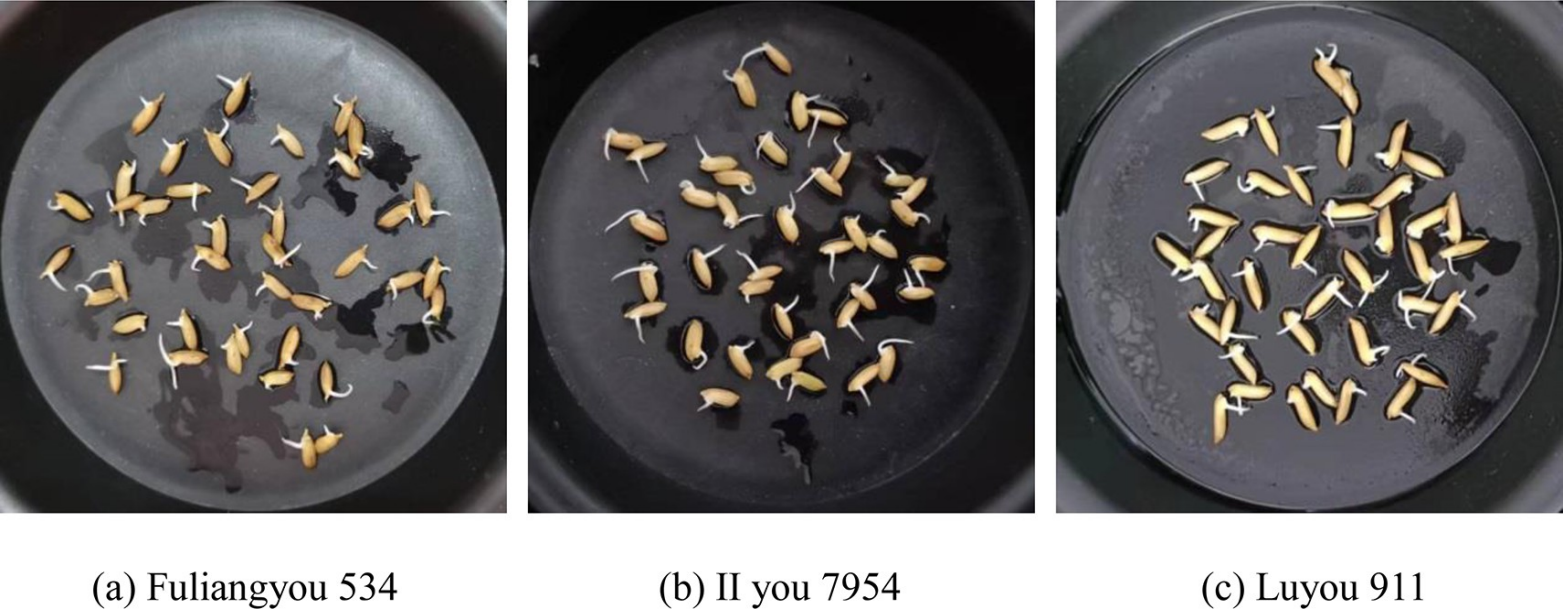
Three rice varieties.

As shown in Fig 9, we used three rice varieties, Fuliangyou 534, II you 7954, and Luyou 911 in the experiments. For convenience, these three varieties are denoted as V1, V2, and V3 respectively.

Absolute and relative errors were used as evaluation indicators in the experiments:

Absoluteerror=|A−B| (9)

$$\mathrm{Relative}\;\mathrm{error}=\frac{\left|A-B\right|}{A}\times 100\%$$ (10)

where *A* represents the true value and *B* represents the predicted value measured by the proposed algorithm.

To verify the accuracy of the proposed grain counting method, we collected 90 grain images of the above-mentioned rice varieties. These images include changes in the number of grains, scale, illumination, and rotation. Table 1 illustrates the mean relative error of grain number for the three rice varieties. As seen in Table 1, the average relative error value of grain count for the 90 images was 1.02% and the standard deviation was 1.26.

**Table 1:** The mean relative error of grain number for the three rice varieties.

Rice varieties	Image number	Mean relative error of grain number (%)	Standard deviation
**V1**	30	0.89	1.13
**V2**	30	1.53	1.57
**V3**	30	0.65	0.80
**Total**	90	1.02	1.26

**Fig 10:**
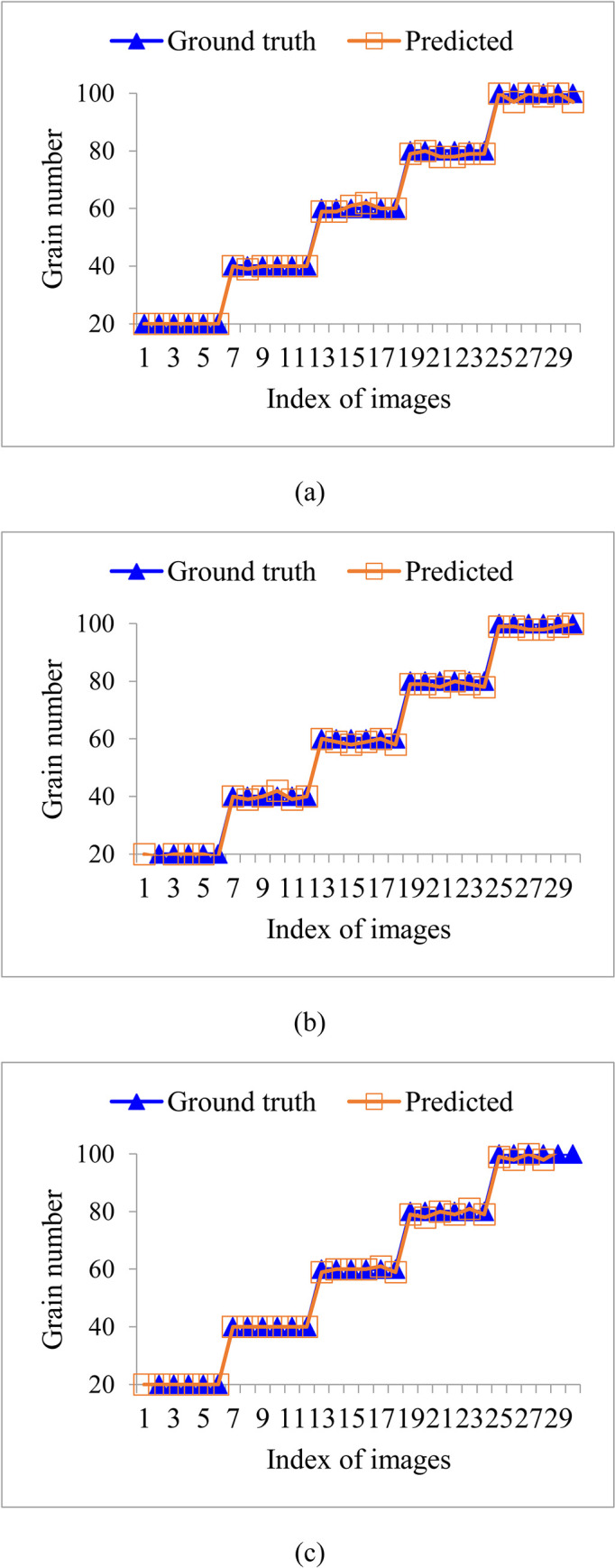
The results of the predicted and true values of grain number for each image in rice varieties V1, V2, and V3: (a) V1, (b) V2, (c) V3.

In addition, Fig 10 shows the results of the predicted and true values of grain number for each image in rice varieties V1, V2, and V3. Each variety includes 30 images. From Fig 10, it can be seen that the predicted values of grain number obtained using the proposed method are close to the true values. And the proposed method is robust for grain varieties and the number of grains contained in the image.

**Fig 11:**
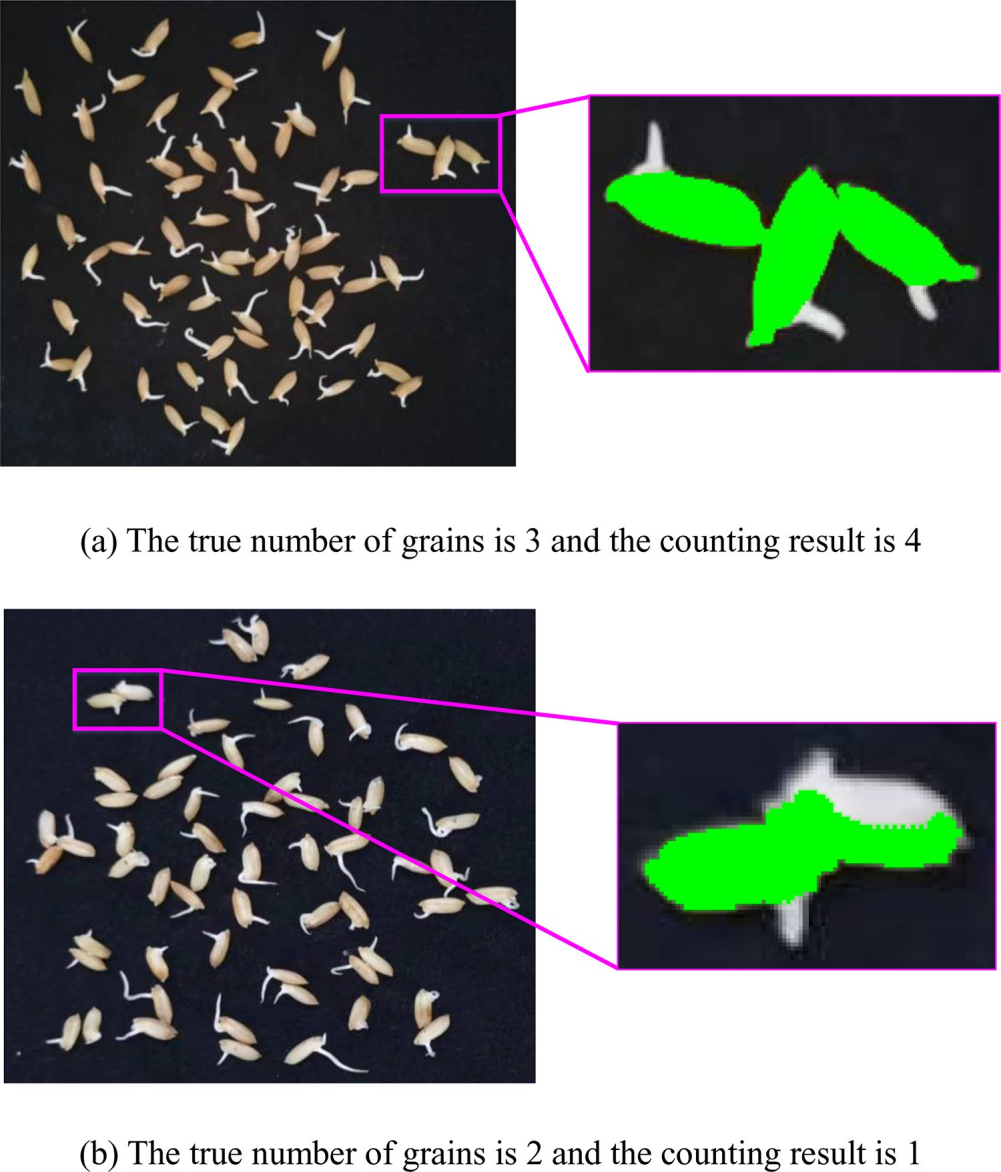
The area of grain connected regions.

We found from the experiments that the counting result may be larger than the true value if there are multiple grains with a larger area than the optimal single grain area. The true number of grains in the connected region shown in Fig 11A is equal to 3. Since the area of each grain in the connected region is larger than the optimal single grain area, the counting result is 4. In addition, the area of the connected area of grains in Fig 11B is small. This is mainly due to the white color of the grains, which makes the grain segmentation wrong. The counting result may be smaller than the true value if there are multiple grains with a smaller area than the optimal single grain area. As shown in Fig 11B, the true number of grains in the connected region is equal to 2. But the counting result is 1.

### Assessment of germination rate of grains

Table 2 illustrates the mean absolute error of germination rate for all images in the three rice varieties. As seen in Table 2, the mean absolute error value of the germination rate for the 90 images was 2.7% and the standard deviation was 2.59.

**Table 2:** The mean absolute error of germination rate for the three rice varieties.

Rice varieties	Image number	Mean absolute error of germination rate (%)	Standard deviation
**V1**	30	3.67	2.97
**V2**	30	2.37	2.45
**V3**	30	2.06	1.99
**Total**	90	2.70	2.59

**Fig 12:**
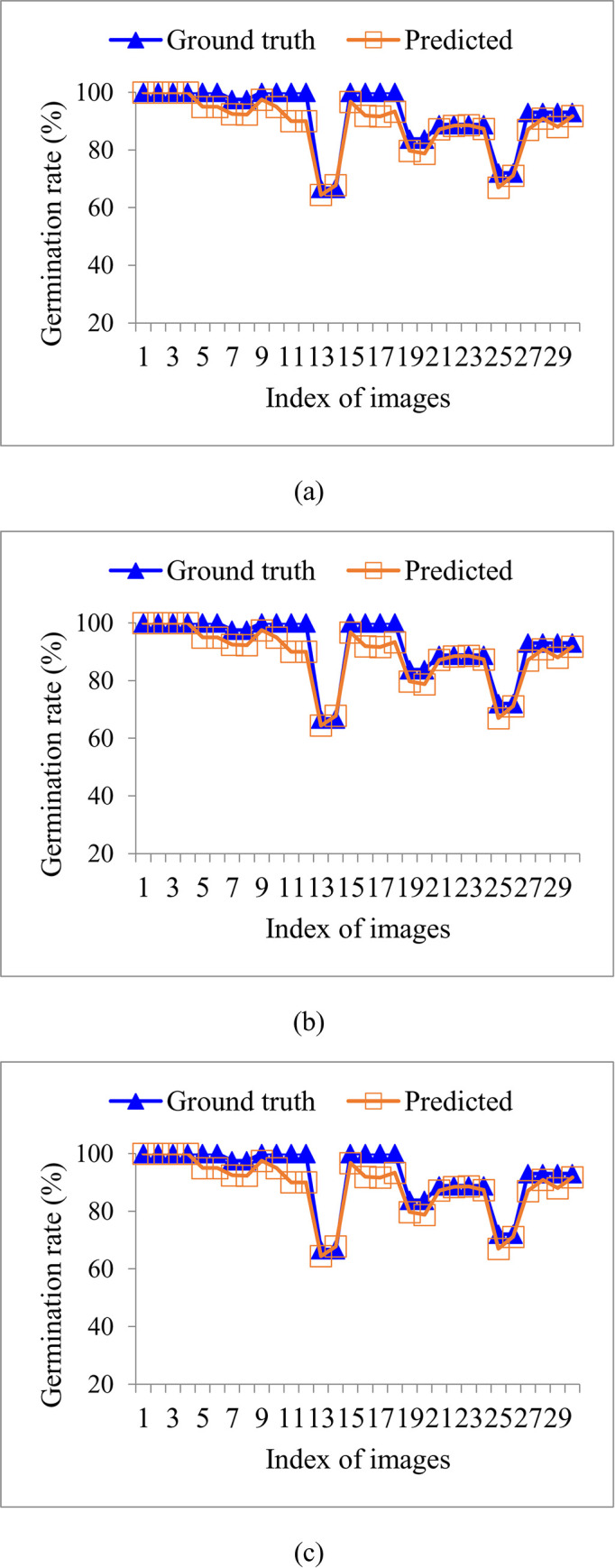
The results of the predicted and true values of germination rate for each image in rice varieties V1, V2, and V3: (a) V1, (b) V2, (c) V3.

In addition, Fig 12 shows the results of the predicted and true values of germination rate for each image in rice varieties V1, V2, and V3. From Fig 12, it can be seen that the predicted values of germination rate obtained using the proposed method are close to the true values.

**Fig 13:**
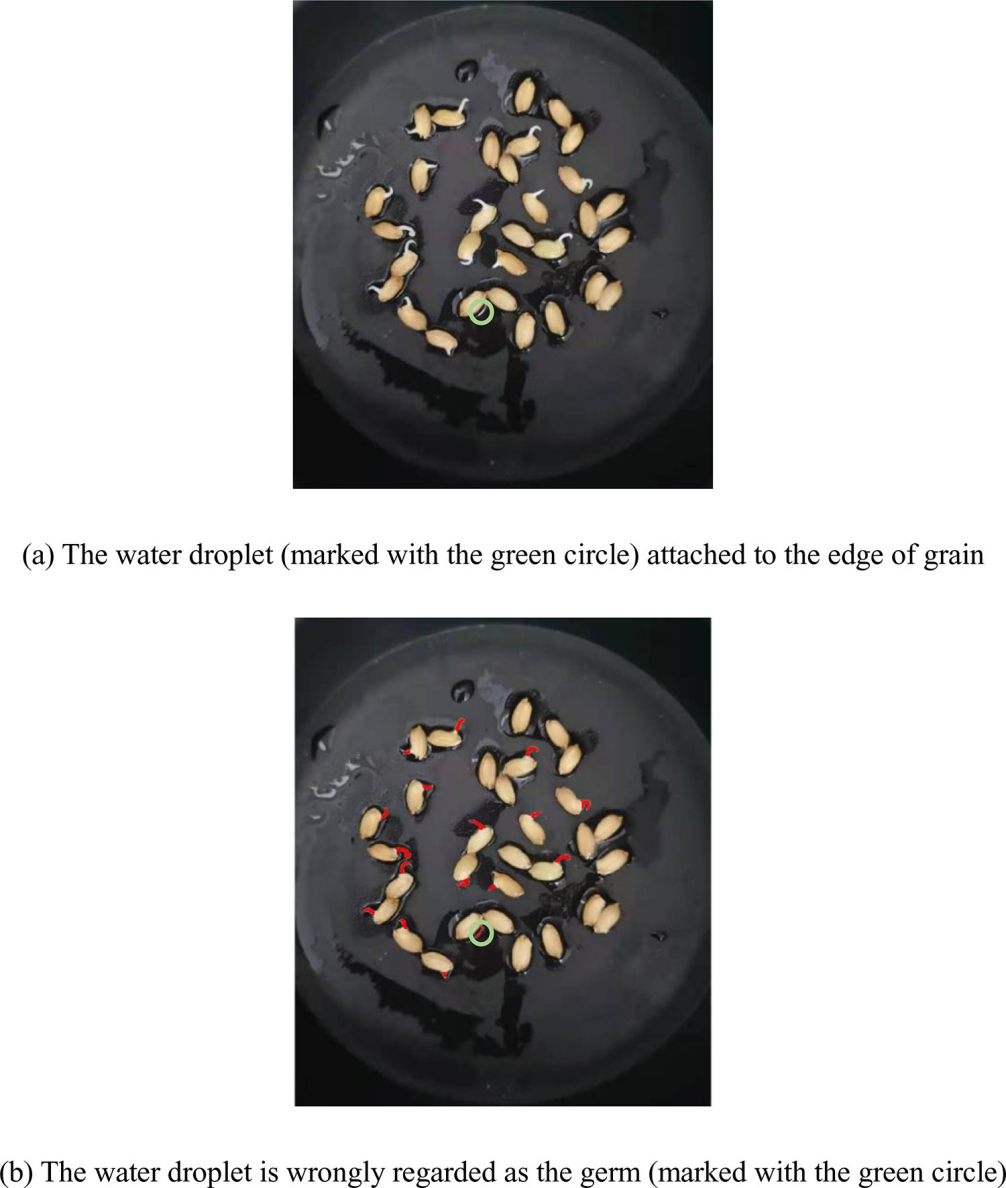
An incorrect example.

As shown in Fig 13A, we found from the experiments that the water droplet attached to the edge of the grain will reflect light, which makes the color of the water droplet appear white. Then the water droplet is wrongly regarded as the germ, as shown in Fig 13B.

## Discussion

**Fig 14:**
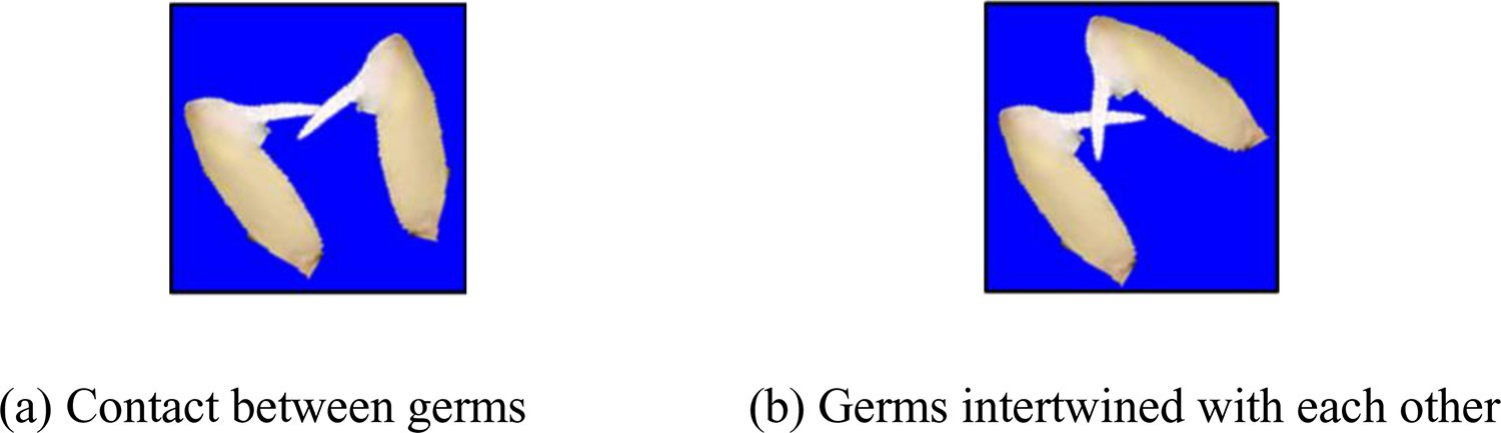
The germs are intertwined or in contact with one another.

In this paper, the germ is considered as a connected region, and the germination rate is calculated by counting the number of germ connected regions. We found that the proposed algorithm would produce incorrect results when there is mutual contact or intertwining between the germs, as shown in Fig 14. This is primarily due to the fact that the germs of two different grains will appear as the same connected region. It is therefore important to avoid any contact or intertwining between the germs when arranging the grains in the container.

**Fig 15:**
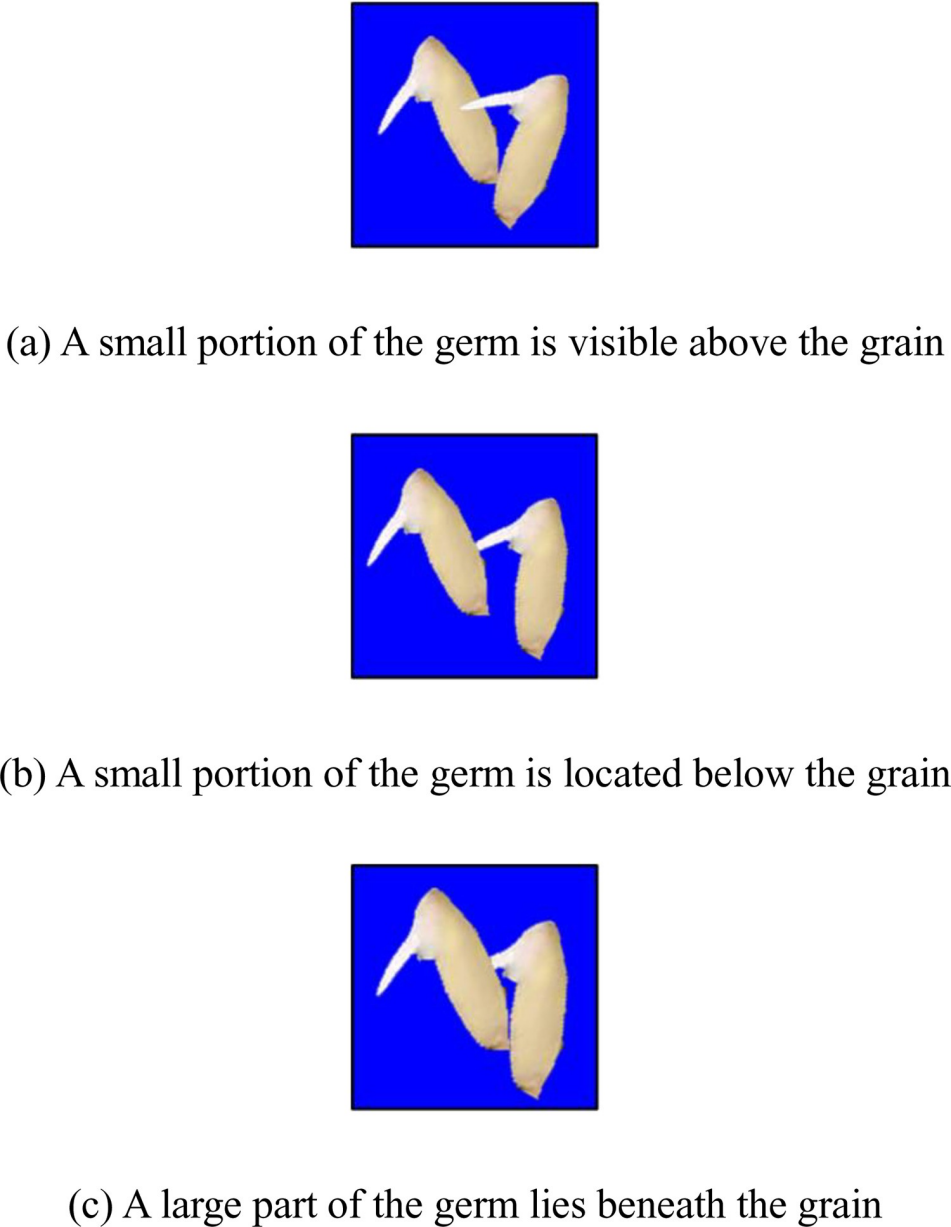
The germ-to-grain contact.

Next, we will discuss germ-to-grain contact. In Fig 15, three cases of germ-to-grain contact and intertwining are illustrated. In Fig 15A, a small portion of the germ is visible above the grain. The area of the grain connected region is smaller than the actual value due to the removal of the germ from the region, which may affect the number of grains. According to Fig 15B, only a small portion of the germ is located below the grain. This has little effect on either the grain number or the germ number. Fig 15C shows that a large part of the germ lies beneath the grain, which will have a significant impact on the number of germs.

**Fig 16:**
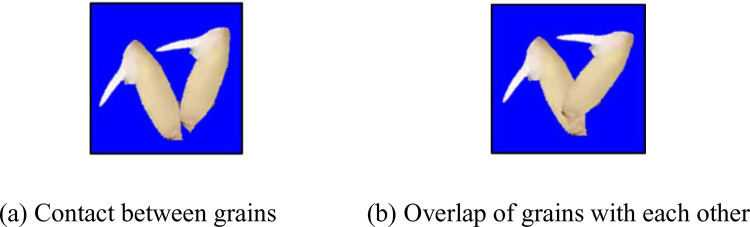
The grains touch or overlap each other.

Then, we will discuss the grain-to-grain contact. In Fig 16A, the mutual touching of grains does not affect the area of the grain connected region, which indicates that the number of grains is not affected by this situation. Fig 16B illustrates that the overlap between grains will affect the area of the grain connectivity area, thereby reducing the number of grains.

Table 3 summarizes the effects of contact or overlap between grains or germs on germination rate. It can be seen from Table 3 that grain-to-grain contact and germ-to-grain contact are allowed, but all other forms of touching or overlapping are prohibited or partially allowed.

**Table 3:** The effects of contact or overlap between grains or germs on germination rate, where ’√’ means allowed and has no effect on germination rate, ’×’ means prohibited and has an effect on germination rate, ’△’ means partially allowed and may have an effect on germination rate.

	Contact	Small amount of overlap or intertwining	Large degree of overlap or intertwining
**Between the grains**	√	Δ	×
**Between the germs**	×	×	×
**Between the grain and the germ**	√	Δ	×

**Fig 17:**
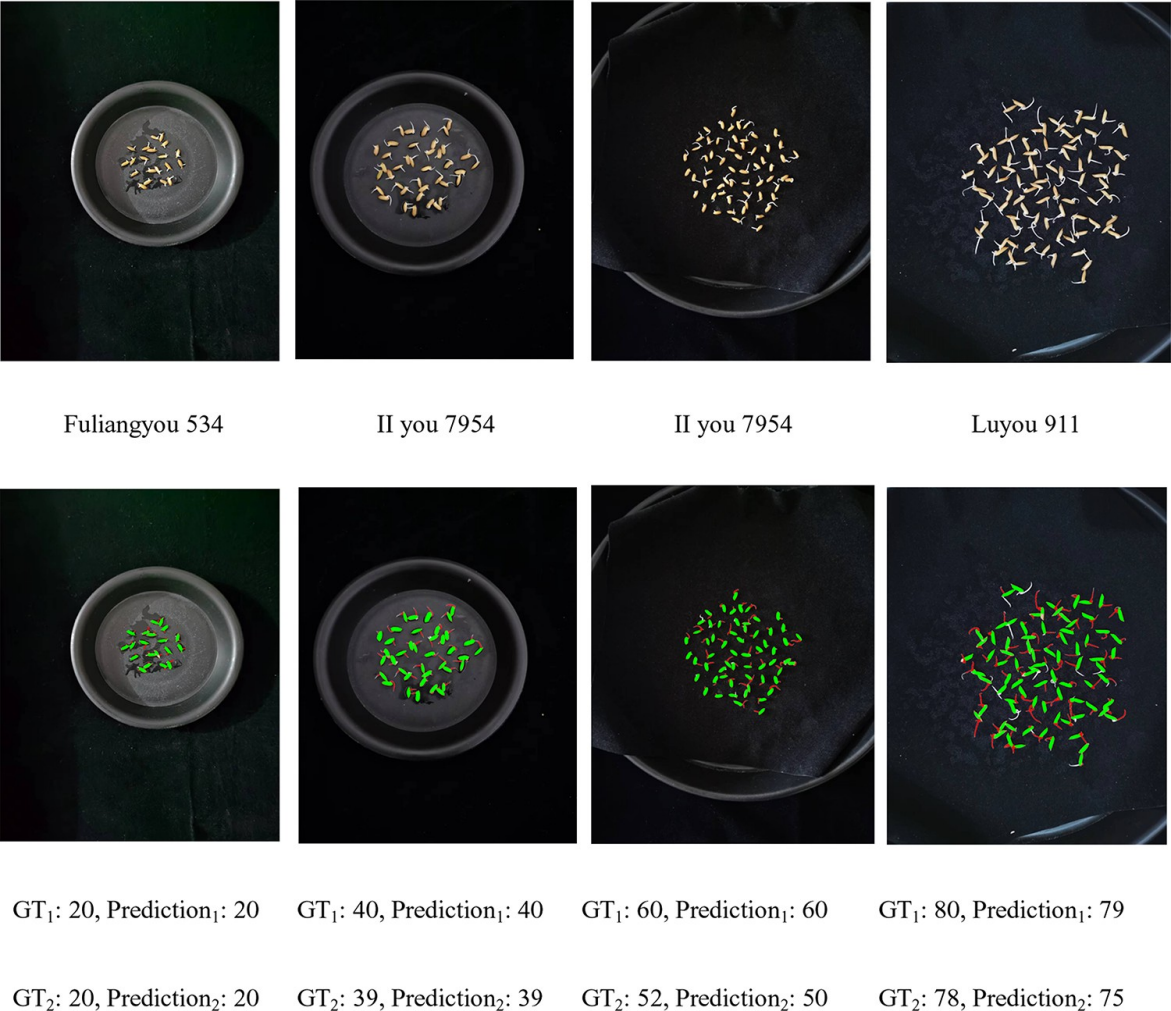
The prediction results of our method on four grain images, where the first row represents the original images, the second row represents the prediction result using our method, green indicates grain, red indicates germ, GT_1_ and prediction_1_ indicate the true and predicted values of grain number, and GT_2_ and prediction_2_ indicate the true and predicted values of germination number, respectively.

As shown in Fig 17, we selected four images out of 90 images that contain varying numbers of grains, 20, 40, 60, and 80. A total of three varieties of Fuliangyou 534, II you 7954, and Luyou 911 can be found within the four images. These four images also differ in their scale, illumination, and rotation. In addition, the proposed method had an average relative error of 1.02% and 2.7% when counting grain and germination number on 90 images, respectively. The application results indicate that the performance of the proposed method is robust to these variations.

## Conclusion

This paper proposes a novel grain germination rate counting algorithm. In this paper, the algorithm proceeds in two steps, counting the number of grains and then counting the number of germs. First, the connected regions where the grains are located are obtained from coarse segmentation to refinement segmentation. The optimal area of each grain is then used to calculate the number of grains per connected region. Finally, the germ number is obtained based on the characteristics of the germ distribution.

We collected 90 grain images to validate the proposed algorithm, including three varieties of Fuliangyou 534, II you 7954, and Luyou 911. These images include changes in the number of grains, scale, illumination, and rotation. The mean absolute error value of the germination rate for the 90 images was 2.7%. The experimental results show that the proposed algorithm can accurately predict the germination rate of grains.

In the future, we plan to explore the dynamics of seed germination uniformity with the proposed algorithm. Moreover, we will optimize the proposed algorithm to be independent of custom color-based thresholds so that the prediction method can be better applied to different crops and light settings.

## Supporting information

S1 Table 30 images of Fuliangyou 534 and the germination detection results. (DOCX)

S2 Table 30 images of II you 534 and the germination detection results. (DOCX)

S3 Table 30 images of Luyou 911 and the germination detection results. (DOCX)

## References

[1] Wu W, Zhou L, Chen J, Qiu Z, He Y. GainTKW: a measurement system of thousand kernel weight based on the android platform. Agronomy. 2018;8(9):178. 10.3390/agronomy8090178.

[2] Geng S, Wu Y, Tian H. Grain Boundary Reconstruction of Metallographical Image Based on Active Contour Model and Mathematical Morphology. Proceedings of the 3rd International Conference on Multimedia and Image Processing 2018. p. 94–98.

[3] Zhang H, Tang Z, Xie Y, Gao X, Chen Q. A watershed segmentation algorithm based on an optimal marker for bubble size measurement. Measurement. 2019;138:182–193. 10.1016/j.measurement.2019.02.005.

[4] Duarte A, Sánchez Á, Fernández F, Montemayor AS. Improving image segmentation quality through effective region merging using a hierarchical social metaheuristic. Pattern Recognition Letters. 2006;27(11):1239–1251. 10.1016/j.patrec.2005.07.022.

[5] Wang W, Paliwal J. Separation and identification of touching kernels and dockage components in digital images. Canadian biosystems engineering. 2006;48:7.

[6] Yang H, Ahuja N. Automatic segmentation of granular objects in images: Combining local density clustering and gradient-barrier watershed. Pattern recognition. 2014;47(6):2266–2279. 10.1016/j.patcog.2013.11.004.

[7] Tan S, Ma X, Mai Z, Qi L, Wang Y. Segmentation and counting algorithm for touching hybrid rice grains. Computers and Electronics in Agriculture. 2019;162:493–504. 10.1016/j.compag.2019.04.030.

[8] Mebatsion H, Paliwal J. A Fourier analysis based algorithm to separate touching kernels in digital images. Biosystems engineering. 2011;108(1):66–74. 10.1016/j.biosystemseng.2010.10.011.

[9] Lin P, Chen Y, He Y, Hu G. A novel matching algorithm for splitting touching rice kernels based on contour curvature analysis. Computers and electronics in agriculture. 2014;109:124–133. 10.1016/j.compag.2014.09.015.

[10] Liu T, Chen W, Wang Y, Wu W, Sun C, Ding J, et al. Rice and wheat grain counting method and software development based on Android system. Computers and Electronics in Agriculture. 2017;141:302–309. 10.1016/j.compag.2017.08.011.

[11] Chen Z, Fan W, Luo Z, Guo B. Soybean seed counting and broken seed recognition based on image sequence of falling seeds. Computers and Electronics in Agriculture. 2022;196:106870. 10.1016/j.compag.2022.106870.

[12] Yao Y, Wu W, Yang T, Liu T, Chen W, Chen C, et al. Head rice rate measurement based on concave point matching. Scientific reports. 2017;7(1):1–11. 10.1038/srep41353. 28128315 PMC5269677

[13] Shadrin D, Menshchikov A, Ermilov D, Somov A. Designing future precision agriculture: Detection of seeds germination using artificial intelligence on a low-power embedded system. IEEE Sensors Journal. 2019;19(23):11573–11582. 10.1109/JSEN.2019.2935812.

[14] Mladenov M, Dejanov M, editors. Application of neural networks for seed germination assessment. Proceedings of the 9th WSEAS international conference on Neural Networks CNN; 2008: Citeseer.

[15] Khoenkaw P, editor An image-processing based algorithm for rice seed germination rate evaluation. 2016 International Computer Science and Engineering Conference (ICSEC); 2016: IEEE.

[16] Pan H, Jiang J, Chen G. TDFSSD: Top-down feature fusion single shot MultiBox detector. Signal Processing: Image Communication. 2020;89:115987. 10.1016/j.image.2020.115987.

[17] Liu W, Anguelov D, Erhan D, Szegedy C, Reed S, Fu C-Y, et al., editors. Ssd: Single shot multibox detector. European conference on computer vision; 2016: Springer.

[18] Girshick R, Donahue J, Darrell T, Malik J, editors. Rich feature hierarchies for accurate object detection and semantic segmentation. Proceedings of the IEEE conference on computer vision and pattern recognition; 2014.

[19] Genze N, Bharti R, Grieb M, Schultheiss SJ, Grimm DG. Accurate machine learning-based germination detection, prediction and quality assessment of three grain crops. Plant methods. 2020;16(1):1–11. doi: 10.1186/s13007-020-00699-x 33353559 PMC7754596

[20] Yang J, Ma Y, Zhang X, Li S, Zhang Y. An initialization method based on hybrid distance for k-means algorithm. Neural computation. 2017;29(11):3094–3117. doi: 10.1162/neco_a_01014 28957026

[21] Zhao W, Ma Y, He X, Huang H, Lin X, Zhang Y, editors. Face Liveness Detection Using Eulerian Video Magnification and SIFT Algorithm. 2020 IEEE International Conference on Artificial Intelligence and Information Systems (ICAIIS); 2020: IEEE.

[22] Tang Q, Wang L, Pan R, Gao W, Lu C. Investigation of fabric shape retention evaluation based on image feature extraction by crease curve fitting. Measurement. 2022;189:110432. 10.1016/j.measurement.2021.110432.

[23] Huang C, Li Z, Liu Y, Wu T, Zeng T. Quaternion-based weighted nuclear norm minimization for color image restoration. Pattern Recognition. 2022;128:108665. 10.1016/j.patcog.2022.108665.

