# Supplementary material for: An automated method for the assessment of the rice grain germination rate
Source: PLoS One. 2023 Jan 3;18(1):e0279934. doi: 10.1371/journal.pone.0279934 (PMC9810190; doi:10.1371/journal.pone.0279934)
Supplement: S3 Table — (DOCX) [file pone.0279934.s003.docx]

Tables S1-S3 shows the 90 images used in the experiment, including Fuliangyuu 534, II you 7954, and Luyou 911 a total of three varieties. The green color in the column of test results indicates the grain and the red color indicates the germ.

**Table S3 30 images of Luyou 911 and the germination detection results**

| No. | Original image | Results of germination rate testing |
| --- | --- | --- |
| 1 | 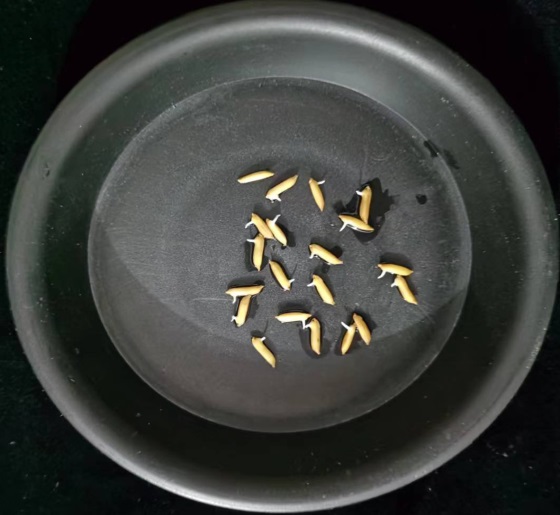 | 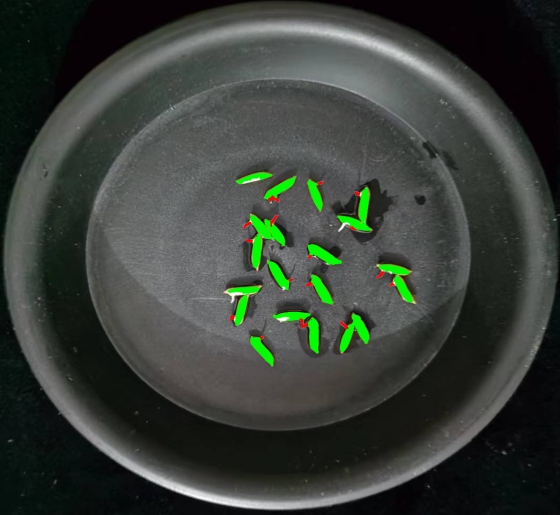 |
| 2 | 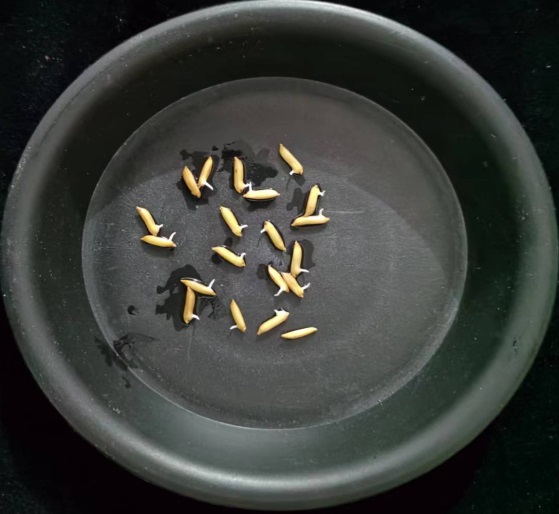 | 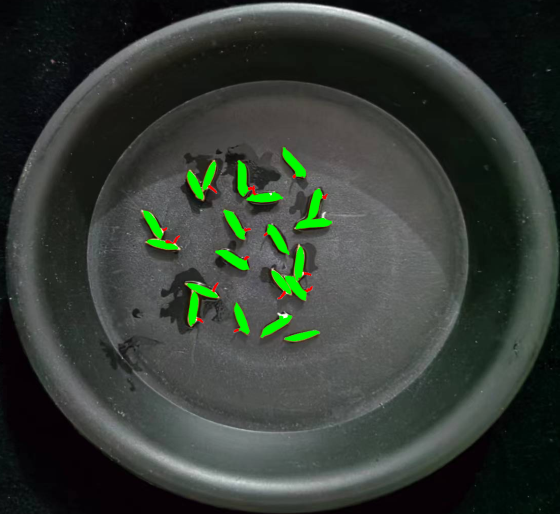 |
| 3 | 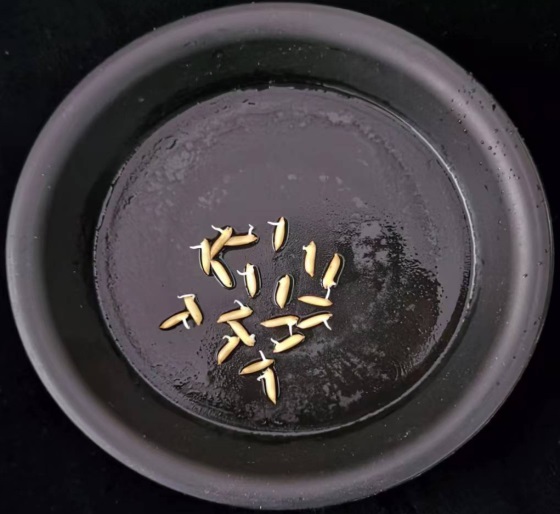 | 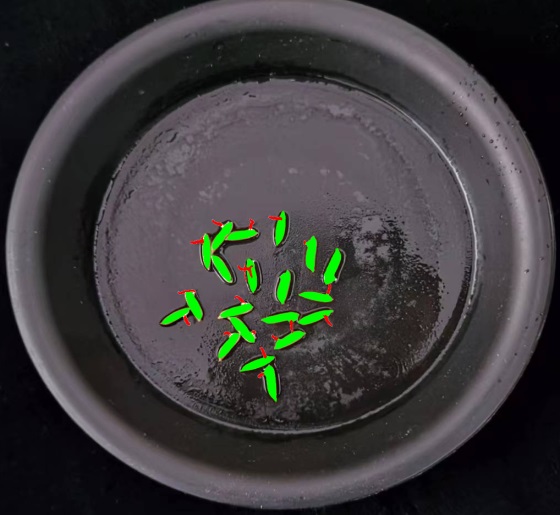 |
| 4 | 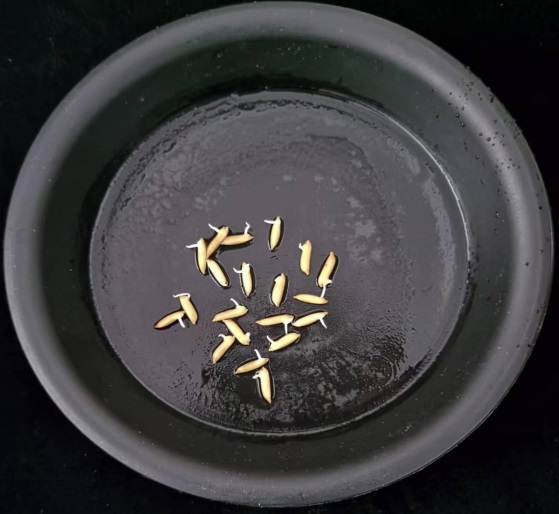 | 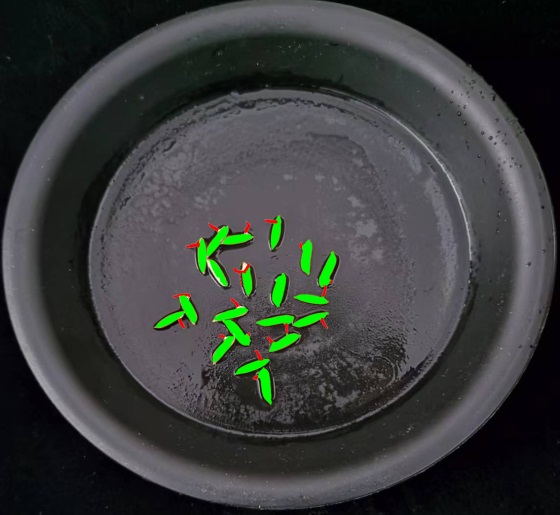 |
| 5 | 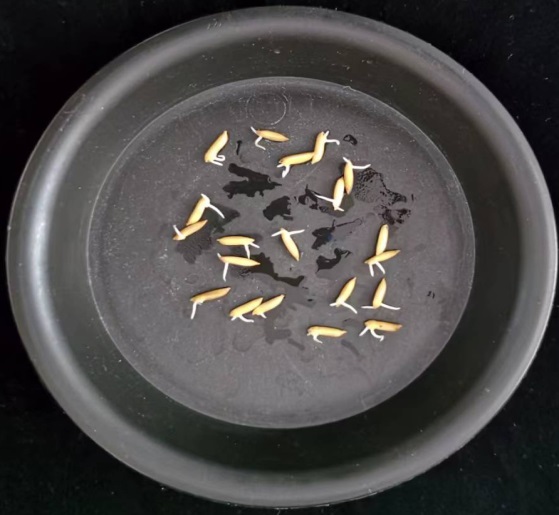 | 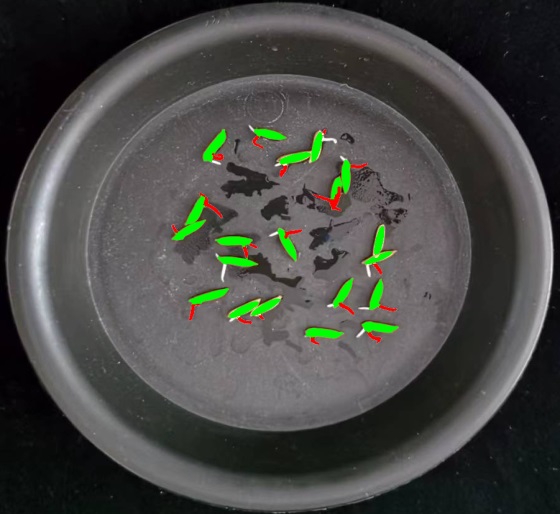 |
| 6 | 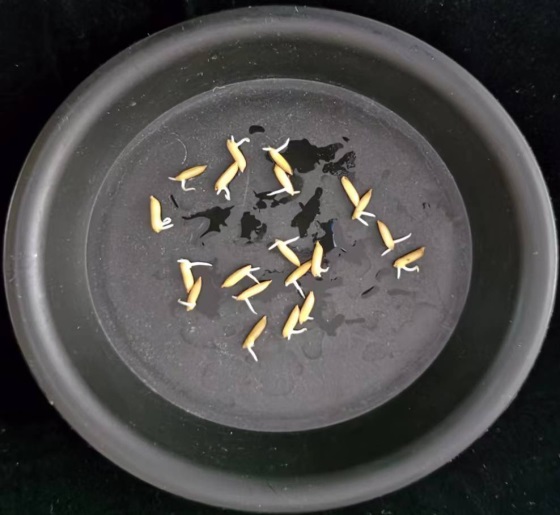 | 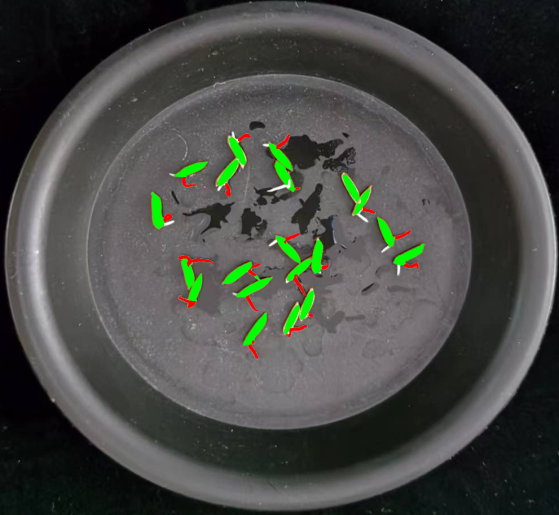 |
| 7 | 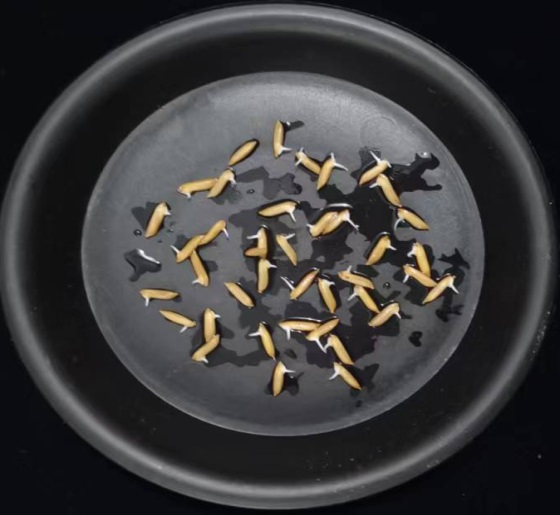 | 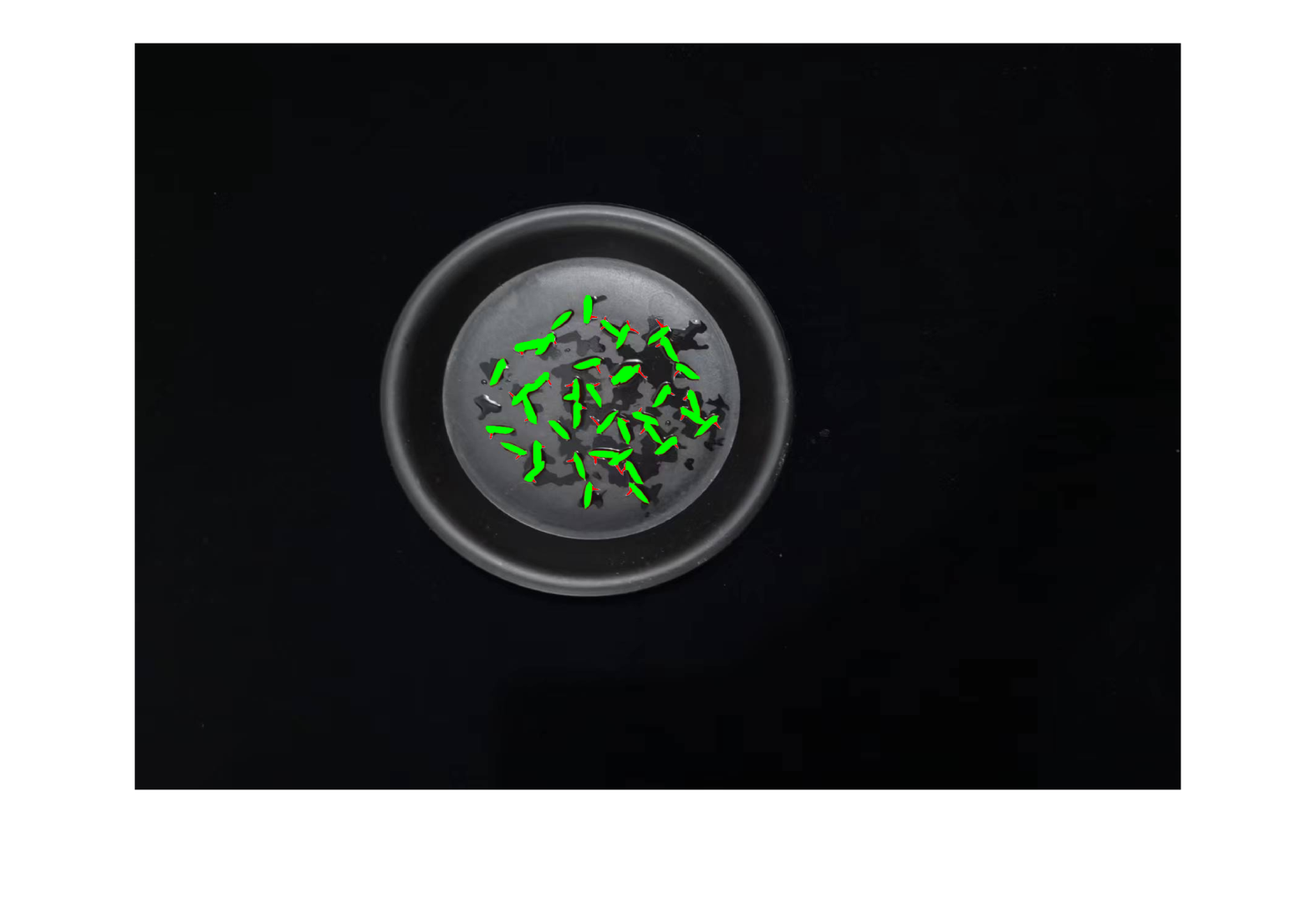 |
| 8 | 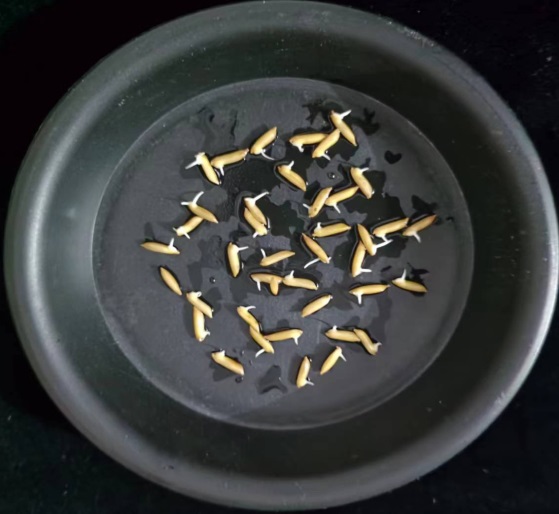 | 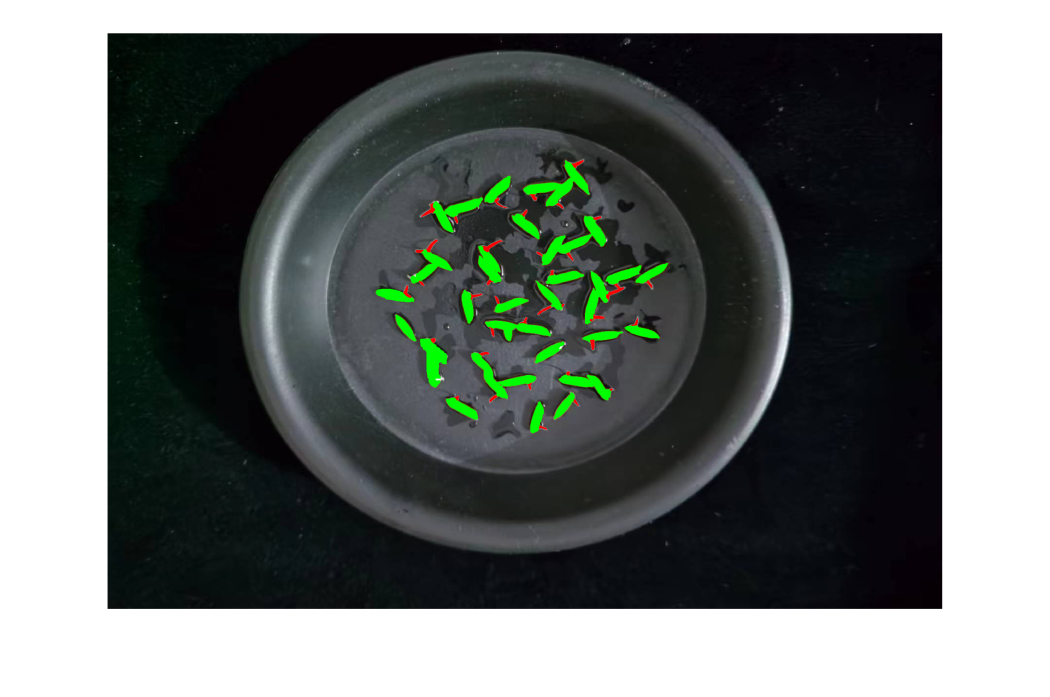 |
| 9 | 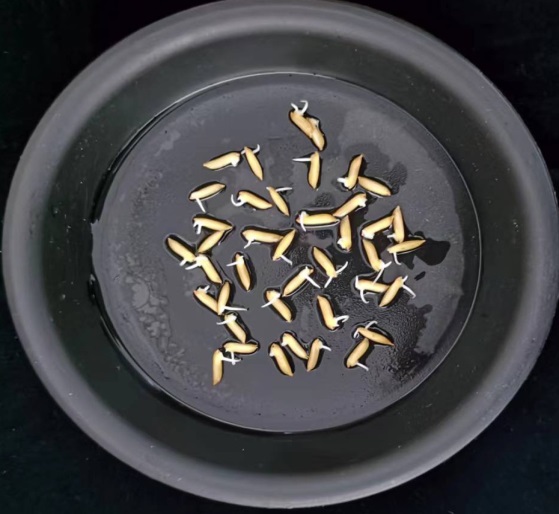 | 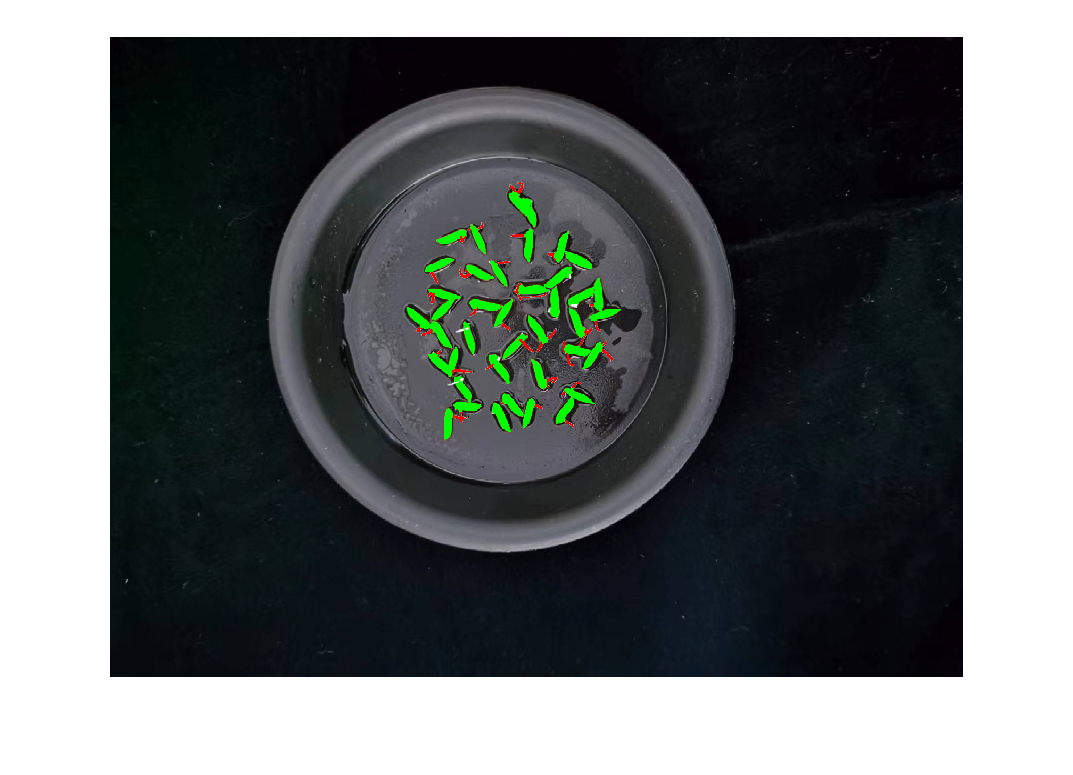 |
| 10 | 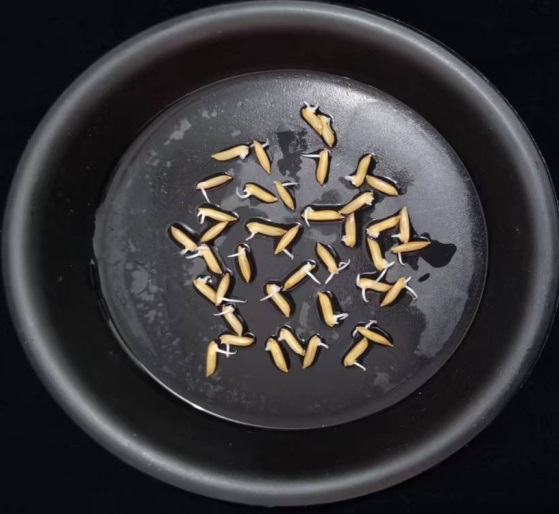 | 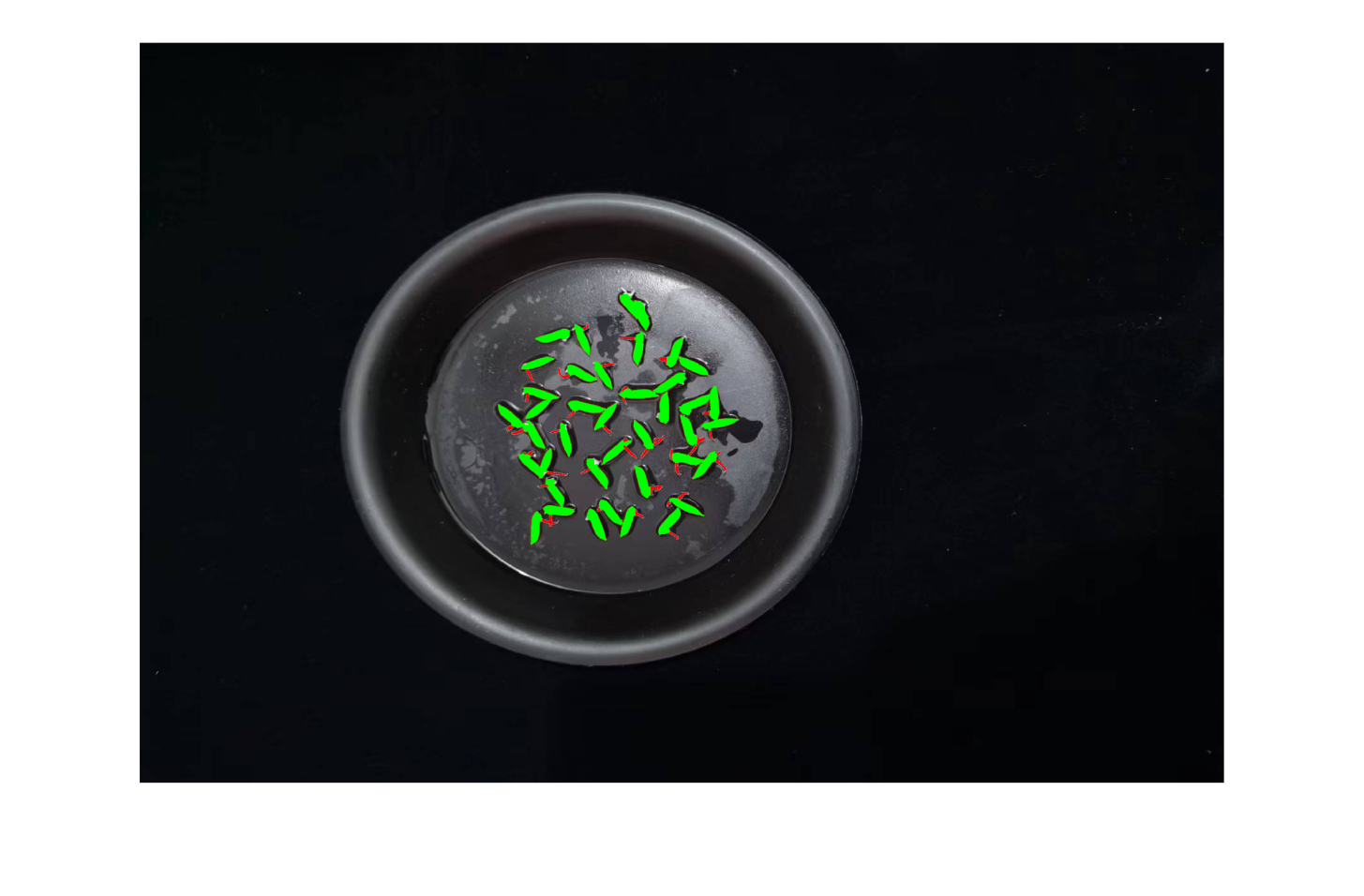 |
| 11 | 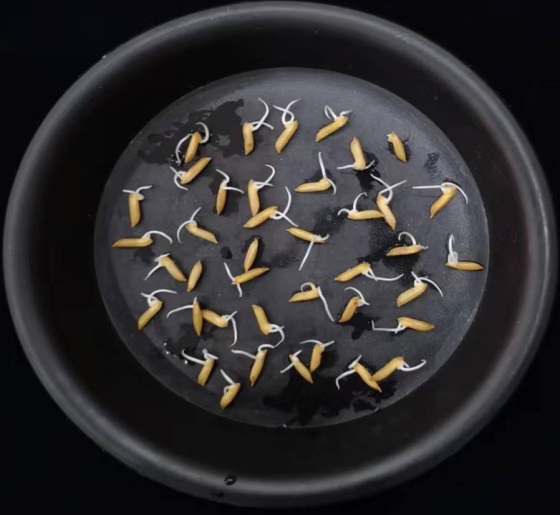 | 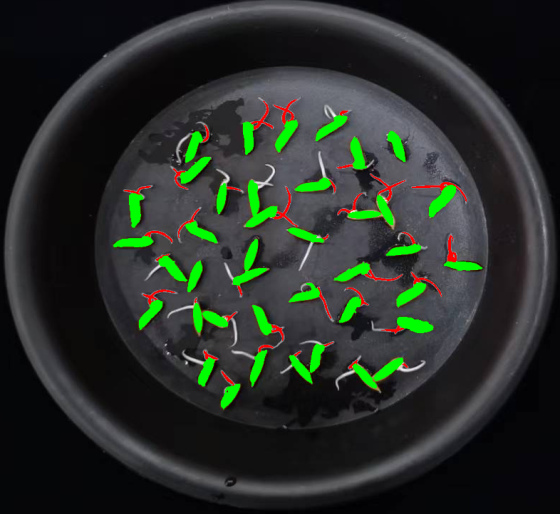 |
| 12 | 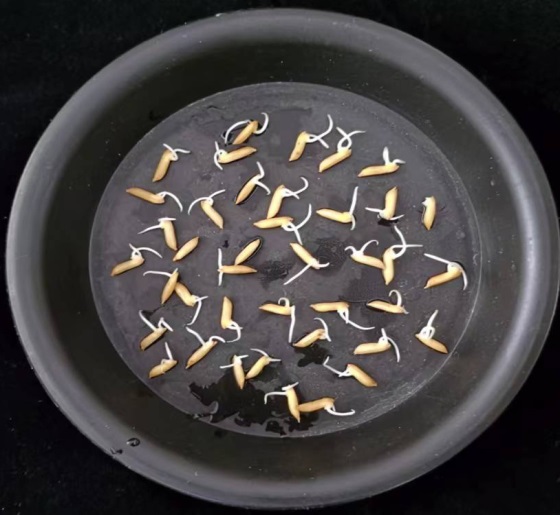 | 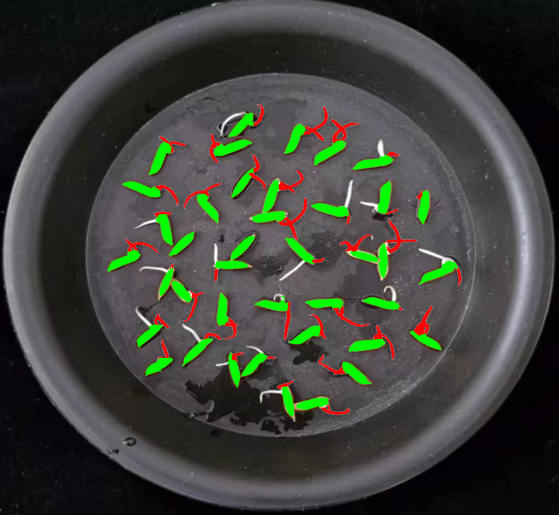 |
| 13 | 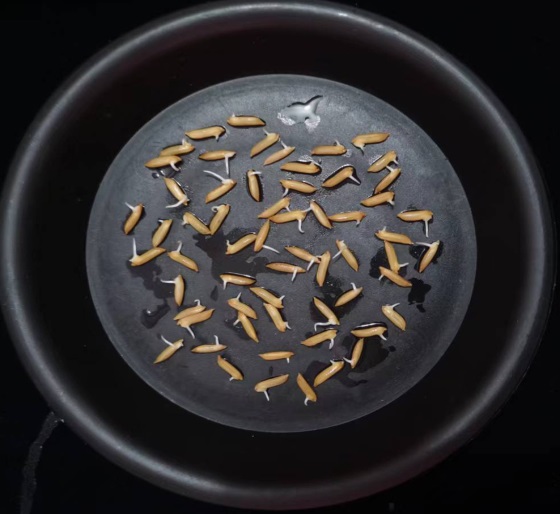 | 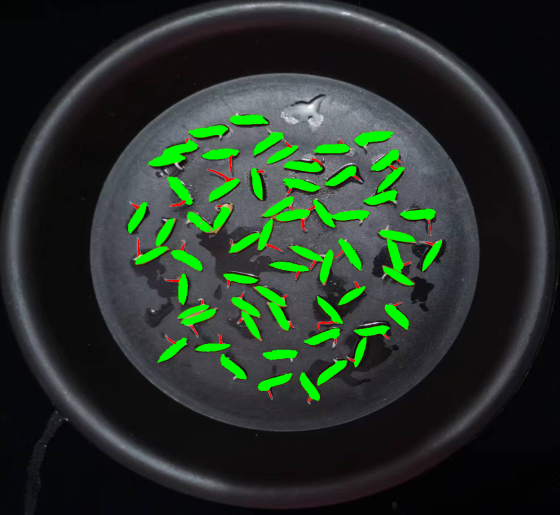 |
| 14 | 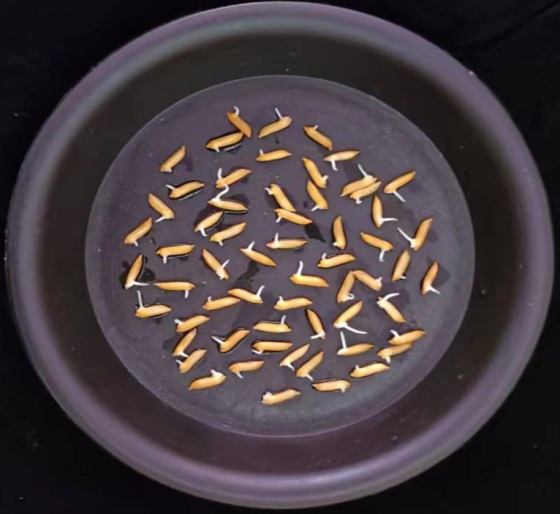 | 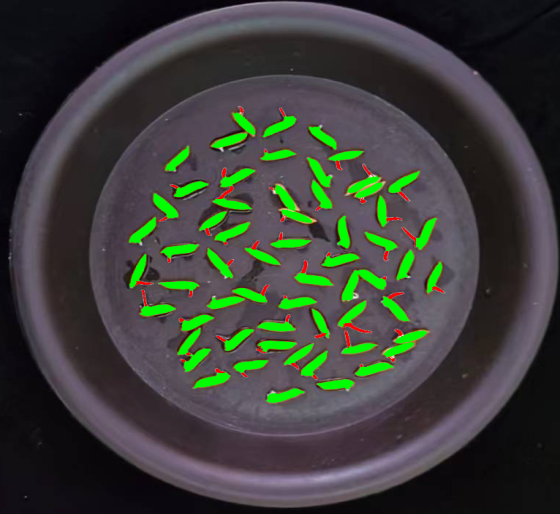 |
| 15 | 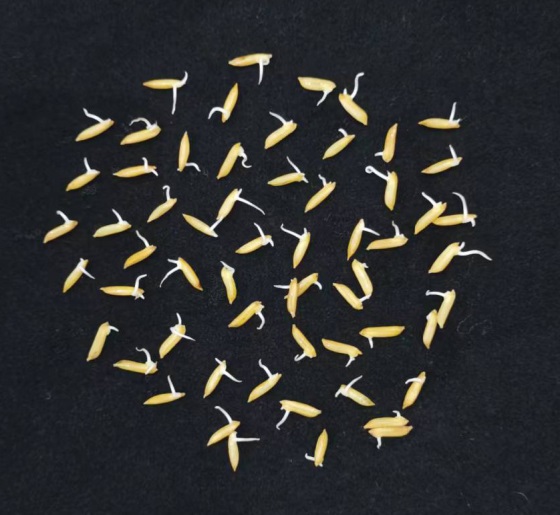 | 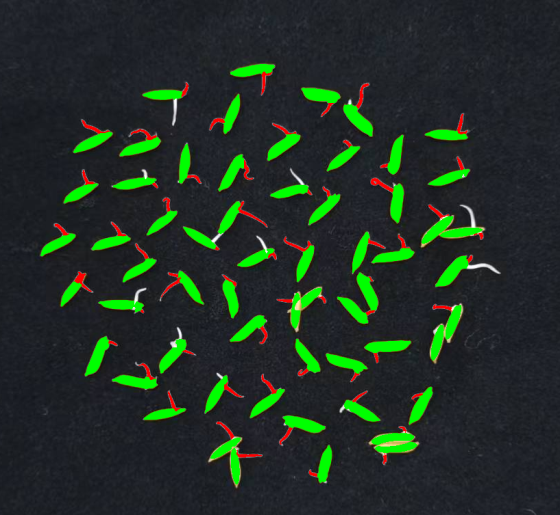 |
| 16 | 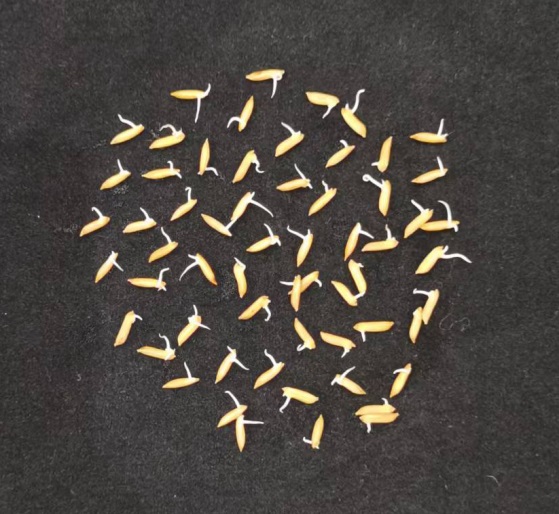 | 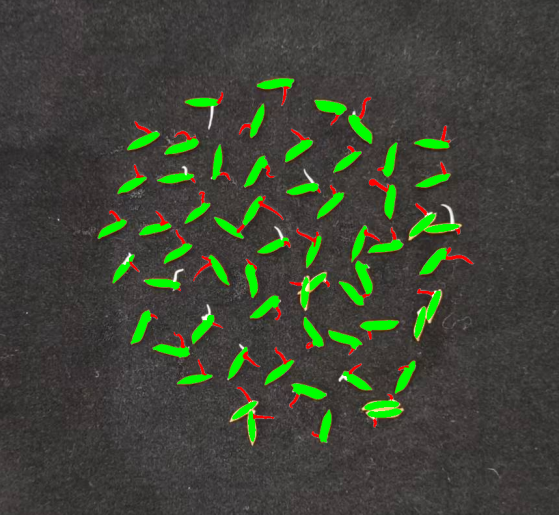 |
| 17 | 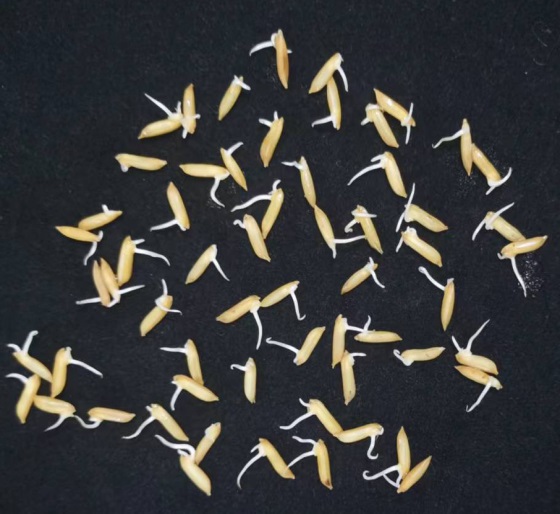 | 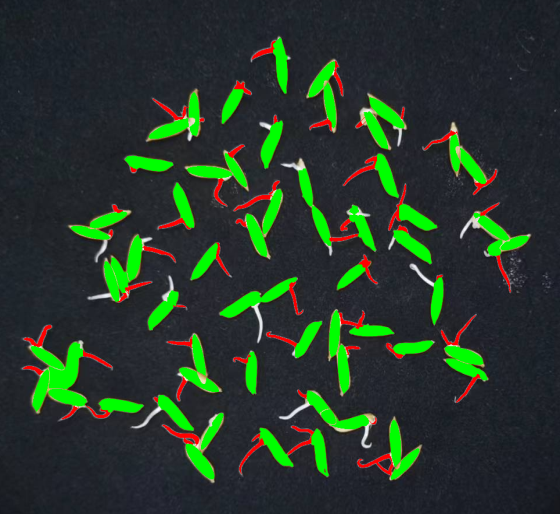 |
| 18 | 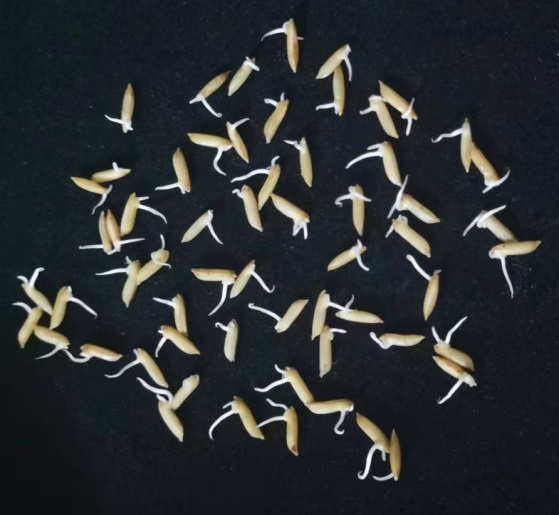 | 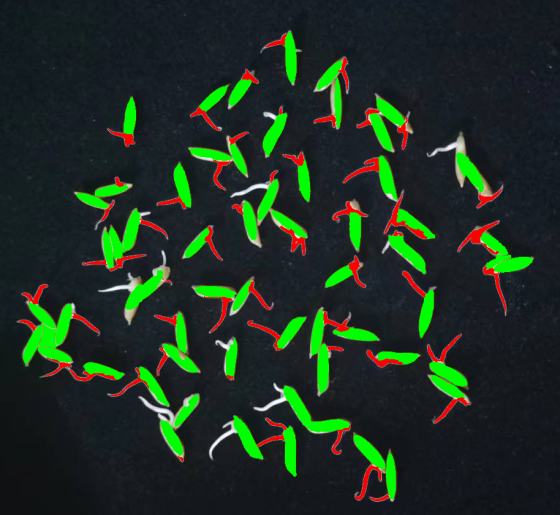 |
| 19 | 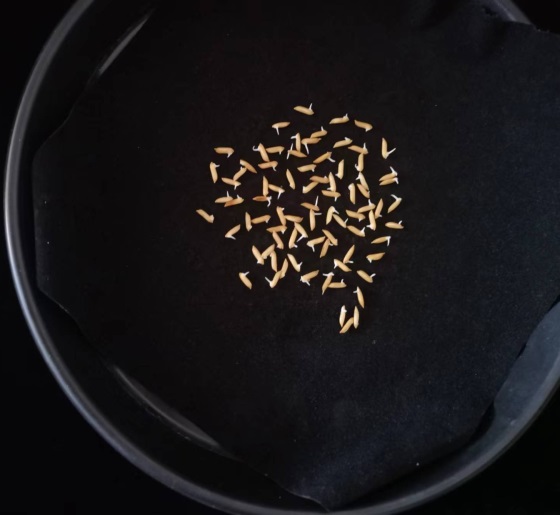 | 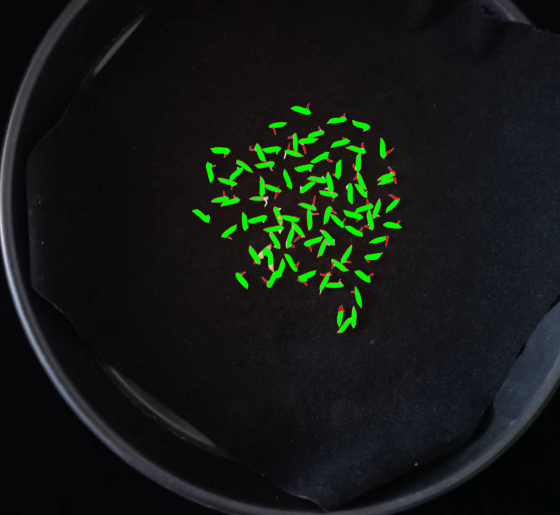 |
| 20 | 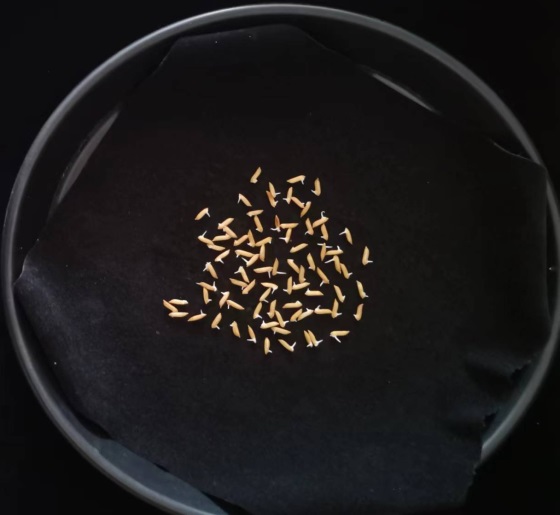 | 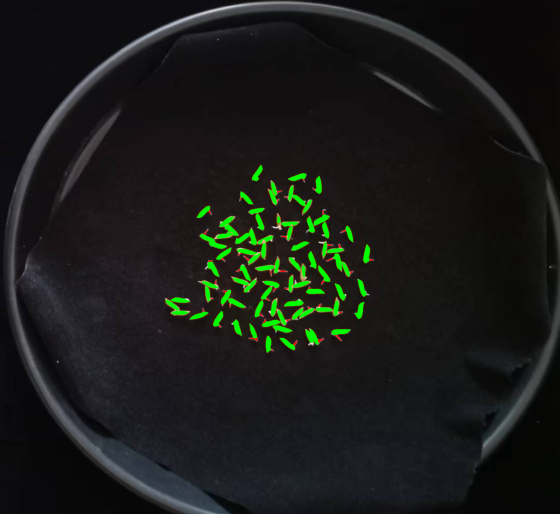 |
| 21 | 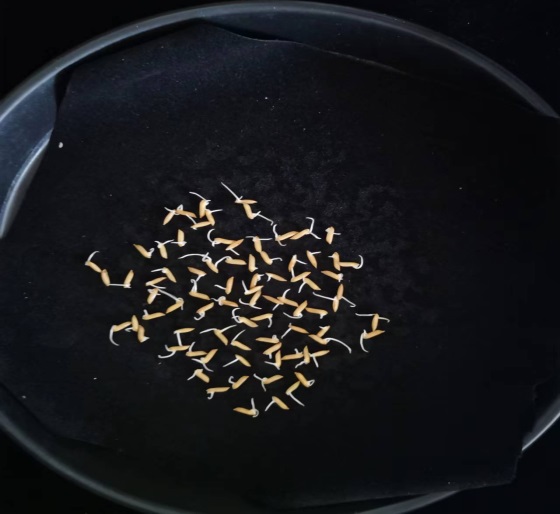 | 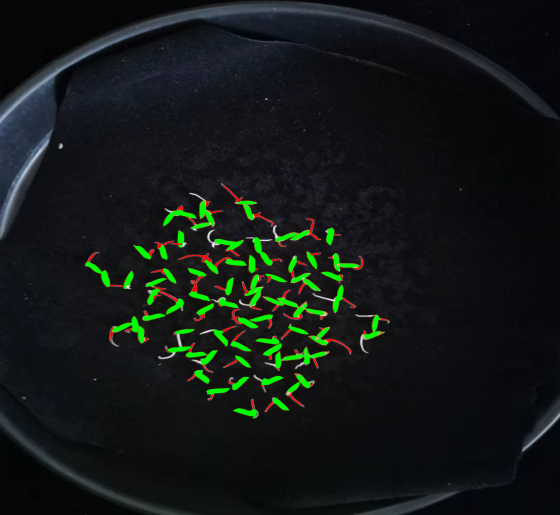 |
| 22 | 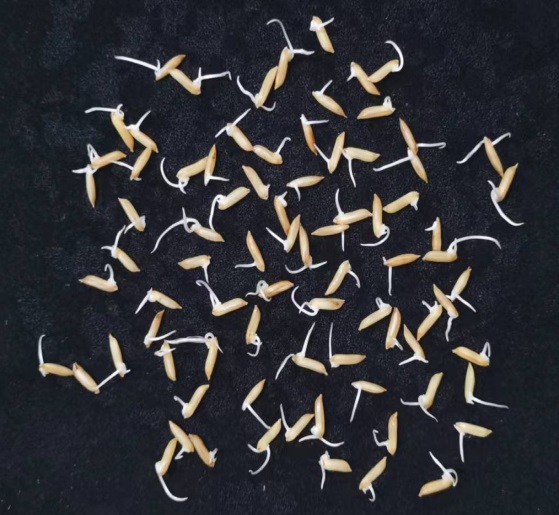 | 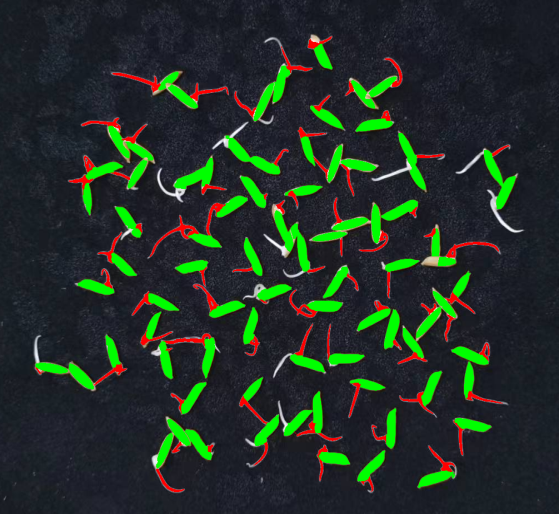 |
| 23 | 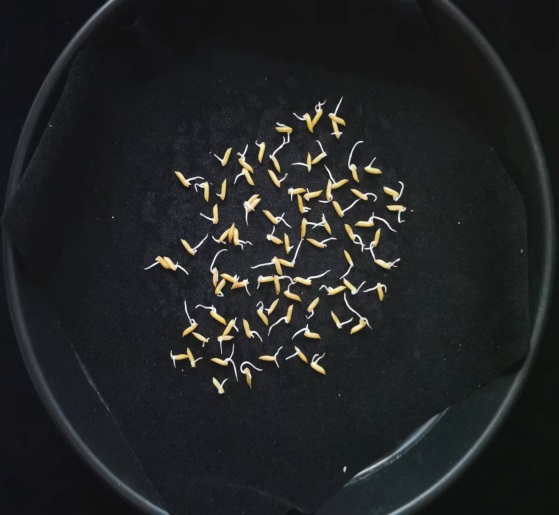 | 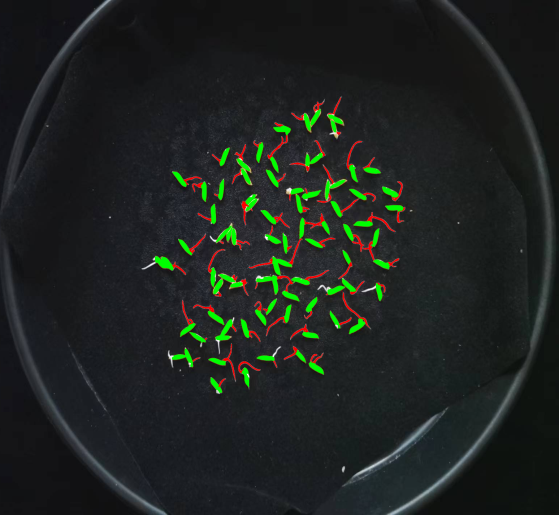 |
| 24 | 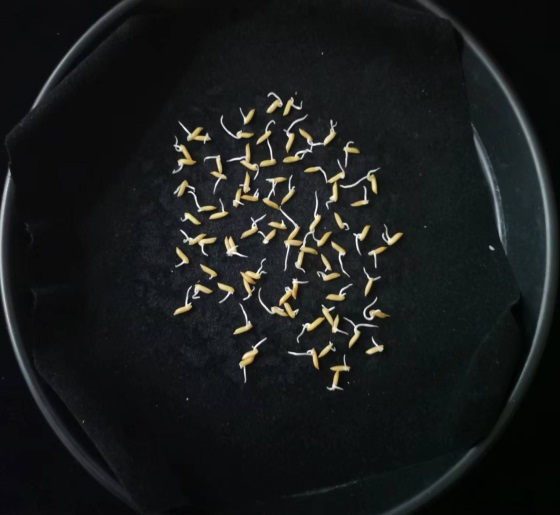 | 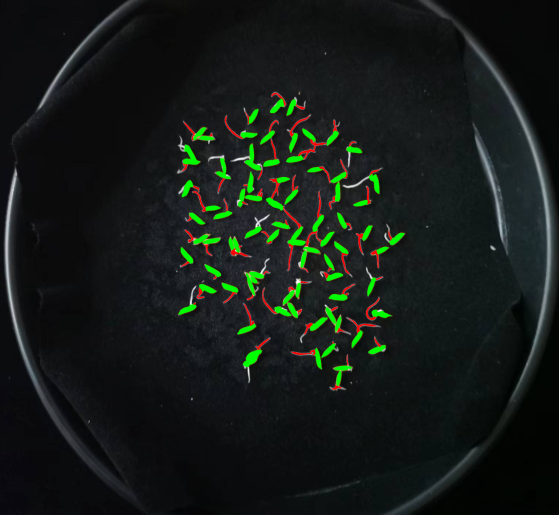 |
| 25 | 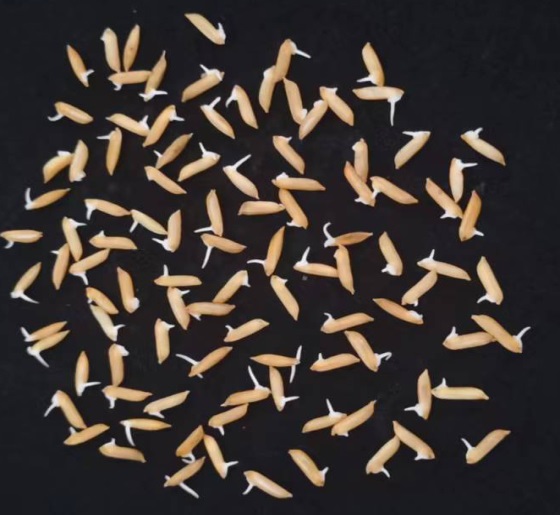 | 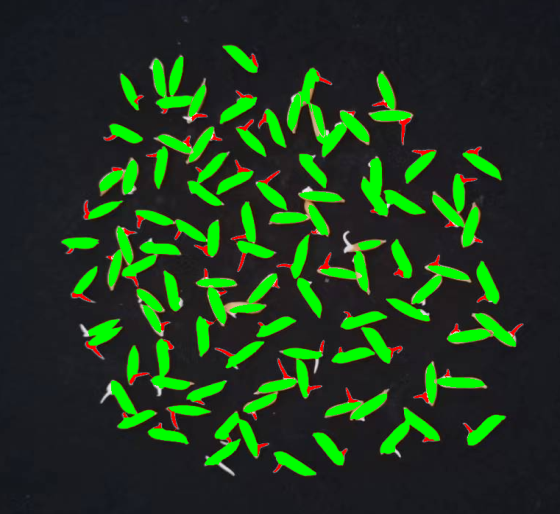 |
| 26 | 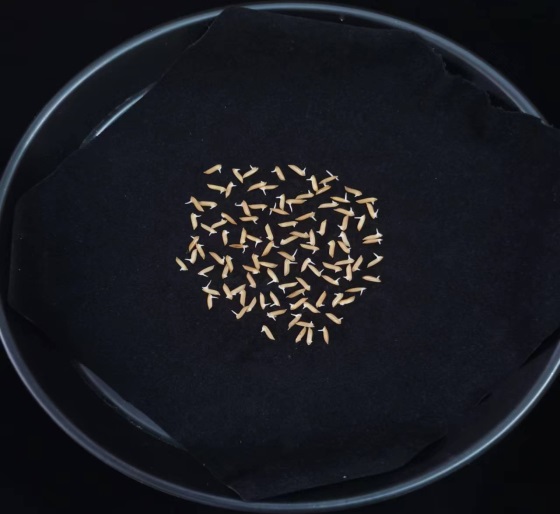 | 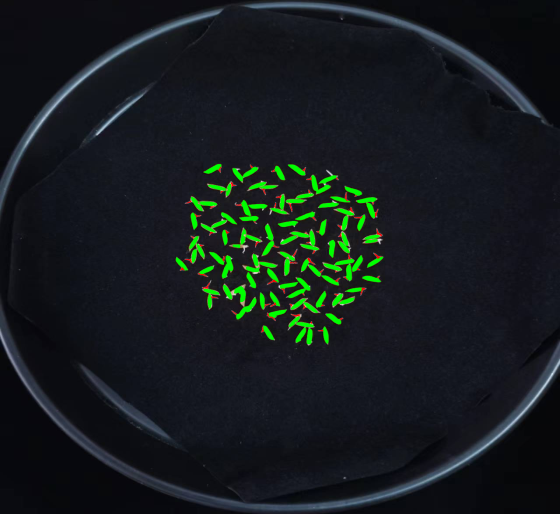 |
| 27 | 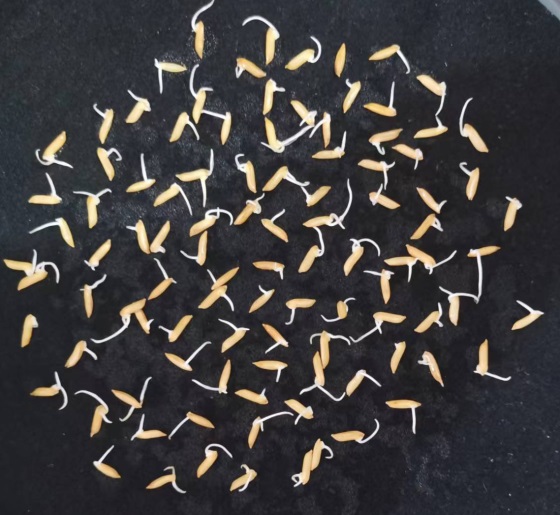 | 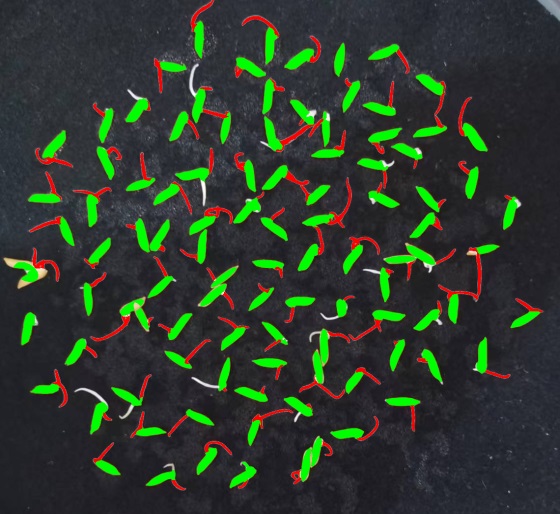 |
| 28 | 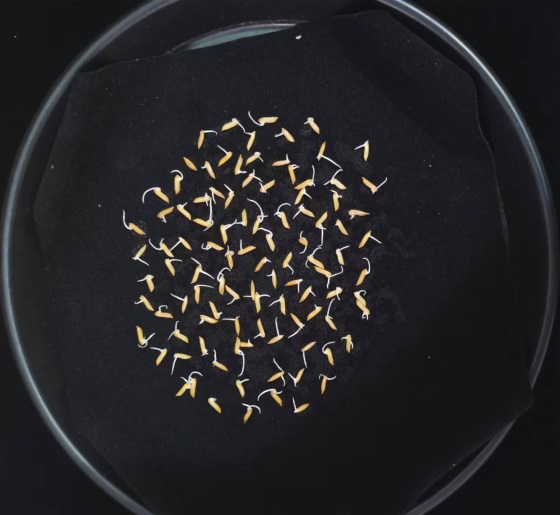 | 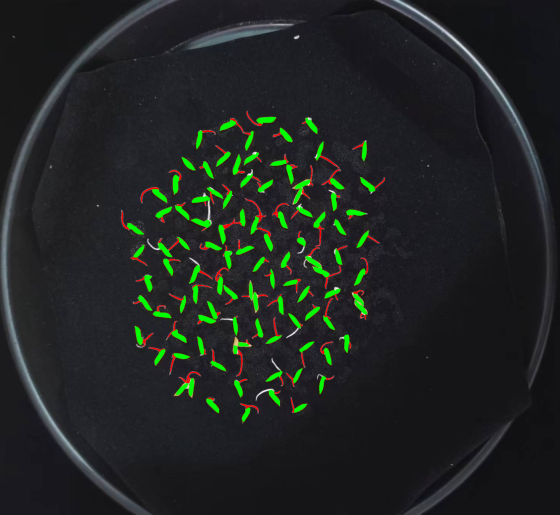 |
| 29 | 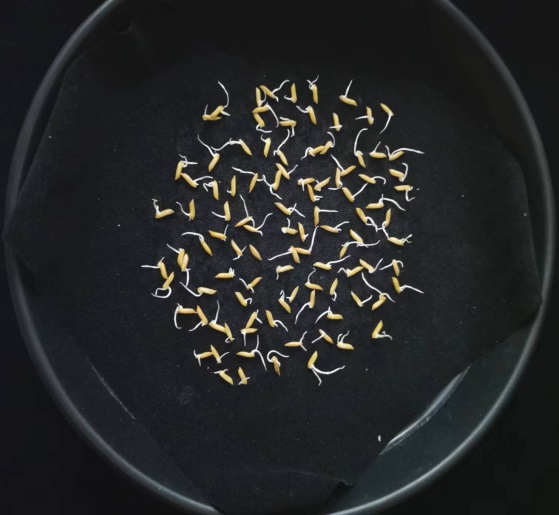 | 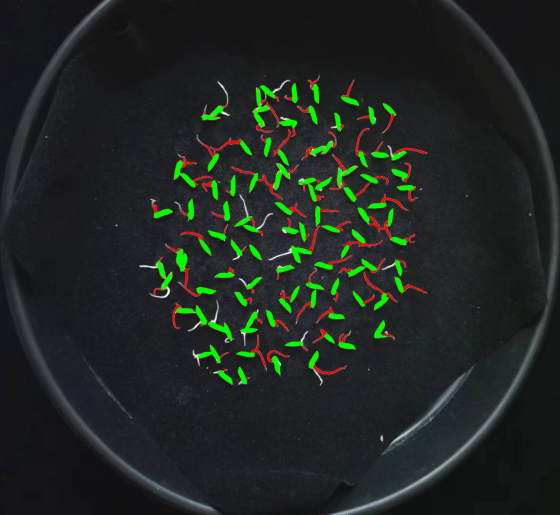 |
| 30 | 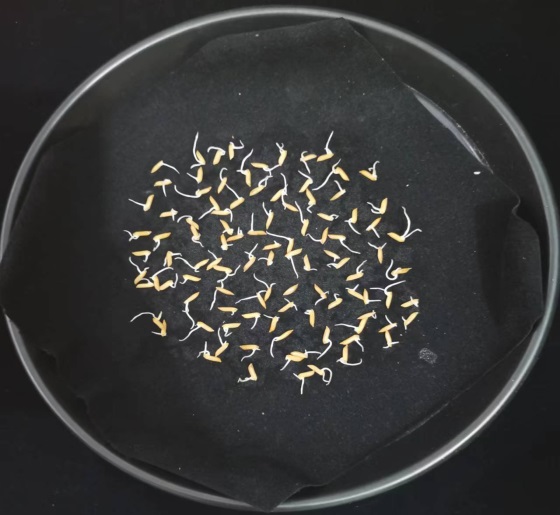 | 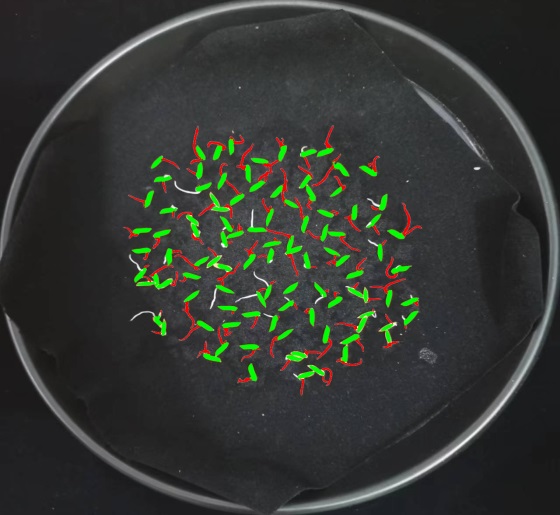 |
